# Trinuclear Cyclometalated Iridium(III) Complex Exhibiting Intense Phosphorescence of an Unprecedented Rate

**DOI:** 10.1021/acs.inorgchem.3c03810

**Published:** 2023-12-28

**Authors:** Marsel Z. Shafikov, Andrey V. Zaytsev, Valery N. Kozhevnikov

**Affiliations:** † Institut für Physikalische und Theoretische Chemie, Universität Regensburg, Universitätsstrasse 31, Regensburg D-93053, Germany; ‡ Department of Applied Sciences, Northumbria University, Newcastle upon Tyne NE1 8ST, U.K.

## Abstract

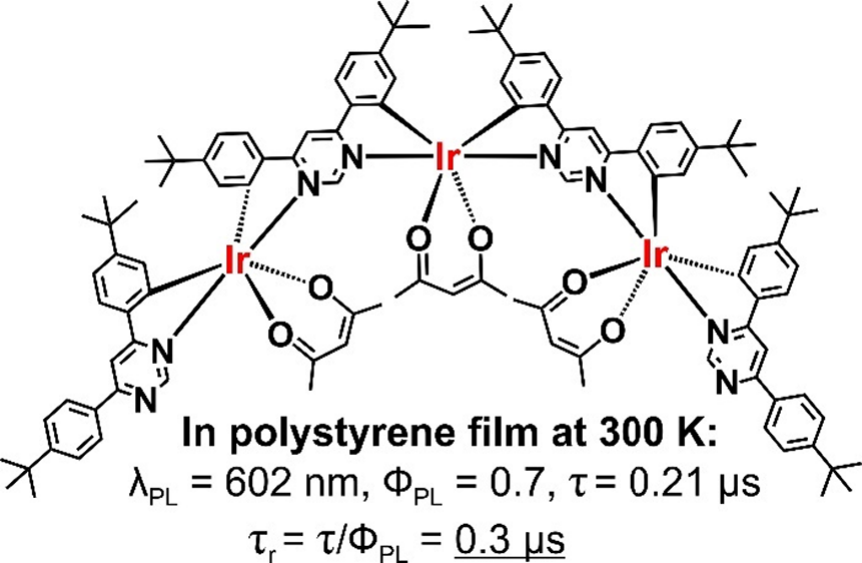

Herein, we present two novel cyclometalated Ir(III) complexes of dinuclear and trinuclear design, **Ir**_**2**_**(dppm)**_**3**_**(acac)**_**2**_ and **Ir**_**3**_**(dppm)**_**4**_**(acac)**_**3**_, respectively, where **dppm** is 4,6-di(4-tert-butylphenyl)pyrimidine ligand and **acac** is acetylacetonate ligand. In both cases, *rac*-diastereomers were isolated during the synthesis. The materials show intense phosphorescence of outstanding rates (*k*_r_ = Φ_PL_/τ) with corresponding radiative decay times of only τ_r_ = 1/*k*_r_ = 0.36 μs for dinuclear **Ir**_**2**_**(dppm)**_**3**_**(acac)**_**2**_ and still shorter τ_r_ = 0.30 μs for trinuclear **Ir**_**3**_**(dppm)**_**4**_**(acac)**_**3**_, as measured for doped polystyrene film samples under ambient temperature. Measured under cryogenic conditions, radiative decay times of the three T_1_ substates (I, III, and III) and substate energy separations are τ_I_ = 11.8 μs, τ_II_ = 7.1 μs, τ_III_ = 0.06 μs, ΔE(II–I) = 7 cm^–1^, and ΔE(III–I) = 175 cm^–1^ for dinuclear **Ir**_**2**_**(dppm)**_**3**_**(acac)**_**2**_ and τ_I_ = 3.1 μs, τ_II_ = 3.5 μs, τ_III_ = 0.03 μs, ΔE(II–I) ≈ 1 cm^–1^, and ΔE(III–I) = 180 cm^–1^ for trinuclear **Ir**_**3**_**(dppm)**_**4**_**(acac)**_**3**_. The determined T_1_ state ZFS values (ΔE(III–I)) are smaller compared to that of mononuclear analogue **Ir(dppm)**_**2**_**(acac)** (ZFS = 210^–1^ cm). Theoretical analysis suggests that the high phosphorescence rates in multinuclear materials can be associated with the increased number of singlet states lending oscillator strength to the T_1_ → S_0_ transition.

## Abstract 2

A trinuclear Ir(III) complex is reported with an unprecedented rate of phosphorescence corresponding to radiative decay time of only 0.3 μs. The properties of the emitting triplet state are investigated in detail with cryogenic steady-state spectroscopy and theoretical methods.

## Introduction

**Chart 1:**
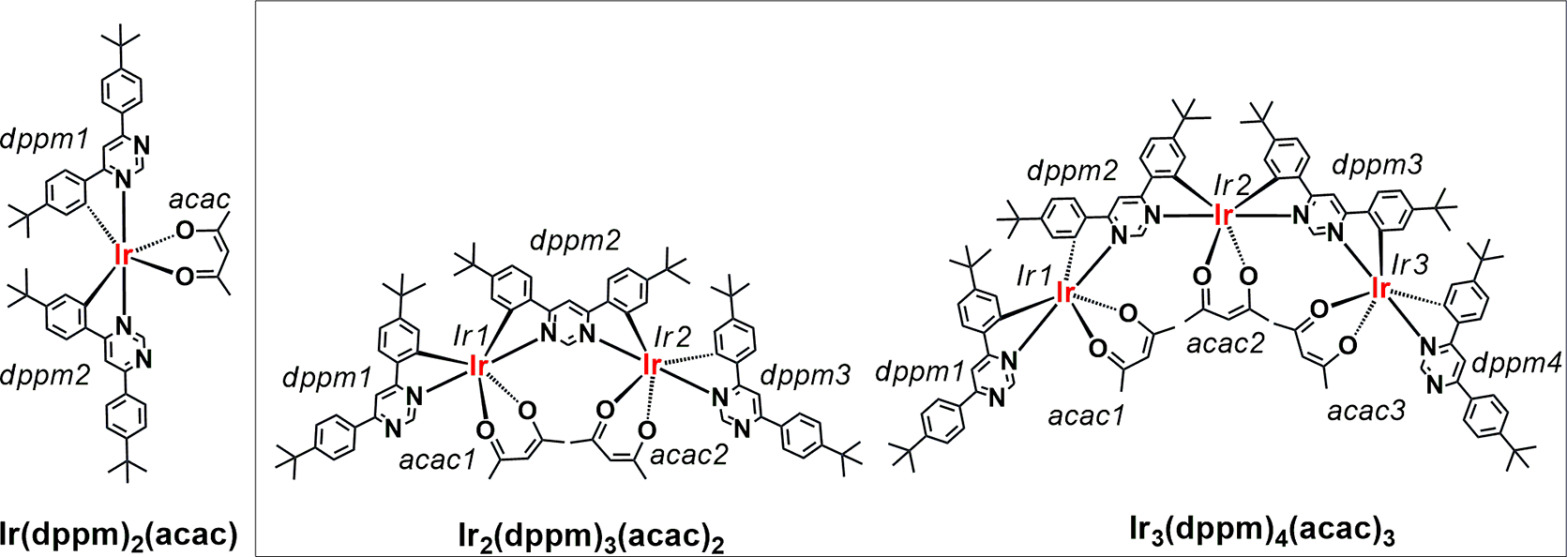
Chemical Structures of **Ir(dppm)**_**2**_**(acac)**, Reported Previously,^21^ and of the Dinuclear **Ir**_**2**_**(dppm)**_**3**_**(acac)**_**2**_ and Trinuclear **Ir**_**3**_**(dppm)**_**4**_**(acac)**_**3**_, Reported in This Work

Transition metal complexes exhibiting phosphorescence—radiative relaxation of the lowest excited triplet state to the singlet ground state (T_1_ → S_0_)—have found application in various fields.^1−11^ This strongly stimulates the research aimed to understand the molecular design principles allowing us to fine-tune their photophysical properties for a particular function. The high rate of phosphorescence, for instance, is a desirable property when fast and efficient utilization of the excited state’s energy is advantageous.^12,13^ For instance, organic light emitting diodes (OLEDs) benefit from the ability of phosphorescent metal complexes to utilize both types of excitons, singlet and triplet, formed in the emitting layer, but the associated triplet–triplet annihilation (TTA) processes, competing with phosphorescence, appear to be a problem limiting the lifespan of the devices. The efficiency of such a deleterious process can be diminished through the enhanced rate of T_1_ → S_0_ phosphorescence. Therefore, materials with enhanced phosphorescence rates and design approaches affording such materials are highly sought after. Complexes of Ir(III) emerged as the most prominent class of materials in terms of the high phosphorescence rate. This is due to the strong metal-induced spin–orbit coupling (SOC) of the lowest excited triplet state T_1_ with singlet states that open the otherwise spin-forbidden T_1_ → S_0_ transition path.^14−16^ A particularly strong SOC of state T_1_ with singlets in Ir(III) complexes is conditioned by a large SOC constant of iridium (ζ = 3909 cm^–1^)^17^ and, importantly, also by the energetical ease of fulfilling the total momentum conservation requirement based on El-Sayed’s rule.^15,16,18^ The latter is due to the (*quasi*)-degeneracy of the occupied t_2g_ symmetry orbitals of d^6^ Ir(III) center (d_xy_, d_xz_, d_yz_) resulting from the splitting of the 5d-orbitals in the (*quasi*)-octahedral ligand field.^19^ As the SOC of state T_1_ with singlet states in Ir(III) complexes is predominantly brought by the metal center, the phosphorescence rate depends on the contribution of the metal to those states. Indeed, a prominent correlation of phosphorescence rate with the extent of metal to ligand charge transfer (MLCT) character of state T_1_ has previously been discussed.^16^ Furthermore, analyzing a number of investigated mononuclear Ir(III) complexes, Yersin *et.al* showed a major trend of radiative decay times (τ_r_ = 1/*k*_r_) of phosphorescence in these materials asymptotically approaching the 1 μs value.^16^ Therefore, the development of design approaches affording phosphorescent materials with radiative decay time significantly shorter than 1 μs is an actual challenge. Presently, only a few exceptional examples of mononuclear Ir(III) complexes with τ_r_ value below 1 μs are known.^20,21^ One of those exceptional complexes has a heteroleptic design (**Ir(dppm)**_**2**_**(acac)** in Chart 1) and utilizes alignment of the two cyclometalating ligands to enhance the oscillator strength of an excited singlet state which, through SOC to the T_1_ state, translates to the enhanced phosphorescence rate.^21^ In recent years, dinuclear molecular design has been shown as advantageous to reach higher phosphorescence rates.^22−28^ In particular, radiative decay times (τ_r_) of below 1 μs were demonstrated as easily attainable for dinuclear Ir(III) complexes with the T_1_ state of strong MLCT character^27−30^ (compare to the cases with T_1_ state of relatively weak MLCT character in refs (31−34)). This renders the multinuclear design of Ir(III) complexes as particularly promising to afford materials with previously unreachable phosphorescence rates.

## Molecular Design and Synthesis

Recently we reported a detailed investigation of a mononuclear Ir(III) complex **Ir(dppm)**_**2**_**(acac)** (Chart 1), comprising derivatives of 4,6-diphenylpyrimidine (**dppm**) and acetylacetonate (**acac**) as ligands, to show its previously unnoticed extraordinarily fast phosphorescence as for a mononuclear complex.^21^ Since **dppm** ligand has two C^N cyclometalating sites, we became interested in multinuclear analogues of **Ir(dppm)**_**2**_**(acac)** with potentially further enhanced phosphorescence rates. However, the octahedral metal center created by bidentate ligands exhibits chirality. Consequently, linking two or more metal centers within a single molecule could generate stereomers, necessitating difficult separation. Previously, we had to overcome this challenge by replacing the bidentate ligands with symmetrical tridentate ones.^24,29,35^ Nevertheless, there are examples of stereoselective synthesis of dinuclear Ir(III) complexes using bidentate ligands.^36^ Usually, the *meso*-product is evidently less favored due to steric interactions arising from the proximity of N^C ligands on adjacent metal centers. Indeed, previously, we noticed that during the synthesis of **Ir(dppm)**_**2**_**(acac)**,^37^ there is the formation of trace amounts of deeply red byproducts, which we suspected to be polynuclear species. We, therefore, decided to carry out the synthesis on a larger scale to isolate and characterize these products. To aid the formation of polynuclear complexes, we used three molar equivalents of the ditopic ligand **dppm** and two molar equivalents of IrCl_3_. After heating under reflux in pure ethoxyethanol, sodium acetylacetonate was added to cleave the dichloro-bridged intermediates. Conveniently, the main product of the reaction, mononuclear **Ir(dppm)**_**2**_**(acac)**, is only moderately soluble in ethanol, and most of it was easily removed by simple filtration. The polynuclear complexes are more soluble and accumulate in the ethanolic filtrate. The desired multinuclear complexes were then chromatographically isolated. Thus, with significant effort, we were able to prepare dinuclear **Ir**_**2**_**(dppm)**_**3**_**(acac)**_**2**_ and trinuclear **Ir**_**3**_**(dppm)**_**4**_**(acac)**_**3**_ (Chart 1), although in very low yields. The low yields and the necessity for chromatographic separation represent primary obstacles that must be addressed in the future optimization of the synthesis. The ^1^H NMR spectrum of the dinuclear **Ir**_**2**_**(dppm)**_**3**_**(acac)**_**2**_ shows one set of signals for the two singly cyclometalated (terminal) **dppm** ligands and one set of signals for the doubly cyclometalated bridging **dppm** ligand. We interpret that, at each coordination center, both cyclometalating (C^N) ligands are *quasi-*equal with respect to the ancillary acac ligand, where each coordinated nitrogen is trans to the coordinated nitrogen of the other dppm ligand, and each coordinated carbon is trans to an oxygen of the **acac** ligand. This configuration was found for the mononuclear **Ir(dppm)**_**2**_**(acac)** where the two dppm ligands appeared to be equal on the ^1^H NMR spectrum. Indeed, this configuration avoids strong trans influence of the two coordinated carbons exerted on each other and is thermodynamically favorable. Due to the possible Λ/Δ-isomerism of the coordination centers, we modeled different stereoisomers of **Ir**_**2**_**(dppm)**_**3**_**(acac)**_**2**_ and **Ir**_**3**_**(dppm)**_**4**_**(acac)**_**3**_. We found that alternation of the Λ and Δ*c*onfigurations between adjacent coordination centers results in steric hindrances between the *tert*-butyl groups of two **dppm** ligands as well as between the two **acac** ligands, which strain the coordination center geometries. Therefore, having only one set of ^1^H NMR signals, we believe that the isolated samples are ΛΛ/ΔΔ-isomers of dinuclear **Ir**_**2**_**(dppm)**_**3**_**(acac)**_**2**_ and ΛΛΛ/ΔΔΔ-isomers of trinuclear **Ir**_**3**_**(dppm)**_**4**_**(acac)**_**3**_. The compositions of **Ir**_**2**_**(dppm)**_**3**_**(acac)**_**2**_ and **Ir**_**3**_**(dppm)**_**4**_**(acac)**_**3**_ are further supported by elemental (C, H, N) and mass-spectrometry analyses. Unfortunately, despite several attempts, we were not able to obtain single crystals suitable for X-ray diffraction analysis for either of the new complexes.

The synthetic details and characterization data are given in the Supporting Information.

## Optical Spectroscopy

The optical spectroscopy measurements were carried out with dilute solutions of **Ir**_**2**_**(dppm)**_**3**_**(acac)**_**2**_ and **Ir**_**3**_**(dppm)**_**4**_**(acac)**_**3**_ in toluene (*c* ≈ 10^–5^ M). The corresponding pertinent data are collected in Table 1. The absorption spectrum of both **Ir**_**2**_**(dppm)**_**3**_**(acac)**_**2**_ and **Ir**_**3**_**(dppm)**_**4**_**(acac)**_**3**_ feature relatively low-intensity bands at the lower energy end (longer wavelengths), presumably due to the significant contribution of charge transfer (CT) excitations, such as metal to ligand charge transfer (d → π*, MLCT), to the character of the associated excited states. Toward the higher energies, the absorption bands gradually gain intensity manifesting the increase of ligand-centered (π → π*, LC) character of the corresponding excited states. These results and assignments are characteristic of intensely emissive Ir(III) complexes and agree well with our DFT calculations (*vide infra*).

**Table 1:** Summary of Key Photophysical Properties of Complexes **Ir(dppm)**_**2**_**(acac)**,^21^**Ir**_**2**_**(dppm)**_**3**_**(acac)**_**2**_ and **Ir**_**3**_**(dppm)**_**4**_**(acac)**_**3**_ Measured as Dilute Toluene Solutions (*c* ≈ 10^–5^ M) and Doped Polystyrene (PS) Films (*c* ≪ 1 wt %)^a^

	**Ir(dppm)_2_(acac)**	**Ir_2_(dppm)_3_(acac)_2_**	**Ir_3_(dppm)_4_(acac)_3_**
absorption λ_max_/nm (ε/M^–1^cm^–1^)	530 (6600), 500 (5900), 426 (11800), 379 (16900), 310 (47100)	576 (10800), 536 (16100), 474 (15500), 427 (22400), 373 (34500), 321 (71600)	580 (21000), 538 (23700), 475 (24260), 425 (31300), 372 (50400), 321 (89500)
photoluminescence in toluene at 300 K			
λ_max_/nm	570	598, 640	600, 645
Φ_PL_/%	80	65	60
τ/μs	0.73	0.27	0.20
τ_r_ = τ/Φ_PL_ /μs	0.91	0.42	0.33
*k*_r_/10^6^ s^–1^	1.10	2.41	3.00
*k*_nr_/10^6^ s^–1^	0.27	1.30	2.00
photoluminescence in toluene at 77 K			
λ_max_/nm	563	590, 638	593, 644
Φ_PL_/%	100	100	90
τ/μs	10.90	2.40	1.40
τ_r_ = τ/Φ_PL_/μs	10.90	2.40	1.56
*k*_r_/10^6^ s^–1^	0.09	0.42	0.64
*k*_nr_/10^6^ s^–1^	<0.003^b^	<0.010^b^	0.064
photoluminescence in PS film at 300 K			
λ_max_/nm	568	600, 642	602, 642
Φ_PL_/%	90	80	70
τ/μs	0.66	0.29	0.21
τ_r_ = τ/Φ_PL_/μs	0.73	0.36	0.30
*k*_r_/10^6^ s^–1^	1.4	2.8	3.3
*k*_nr_/10^6^ s^–1^	0.15	0.69	1.4

a The absorption spectrum was measured in toluene under ambient conditions. The ambient temperature (300 K) emission decay time and quantum yield values were measured for degassed toluene solution and PS films under nitrogen. The radiative decay rates are calculated as *k*_r_ = Φ_PL_/τ; the non-radiative decay rates are calculated as *k*_nr_ = (1−Φ_PL_)/τ; the radiative decay times are calculated as τ_r_ = τ/Φ_PL_ = 1/*k*_r_.

b These values are estimated assuming 3% error in the emission quantum yield value, e.g., assuming Φ_PL_ = 0.97 (97%).

**Figure 1:**
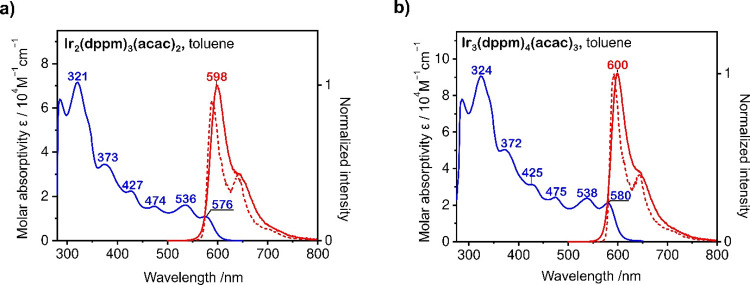
Absorption spectrum (blue trace) and emission spectrum at *T* = 300 K (solid red line) and at *T* = 77 K (dashed red line) measured for dilute toluene solutions (*c* = 10^–5^ M) of (a) **Ir**_**2**_**(dppm)**_**3**_**(acac)**_**2**_ and (b) **Ir**_**3**_**(dppm)**_**4**_**(acac)**_**3**_.

Both complexes, **Ir**_**2**_**(dppm)**_**3**_**(acac)**_**2**_ and **Ir**_**3**_**(dppm)**_**4**_**(acac)**_**3**_, exhibit intense red photoluminescence, with the maxima at ca. 598 nm and ca. 600 nm, respectively, as measured for dilute toluene solution and doped polystyrene (PS) film samples under ambient conditions (Figure 1 and Table 1). In both cases, the emission spectrum strongly overlaps with the lowest energy absorption band (Figure 1). Tentatively assigning the observed emission to T_1_ → S_0_ phosphorescence, similarly to that in mononuclear **Ir(dppm)**_**2**_**(acac)**,^21^ such an spectral overlap is evident of a relatively small energy separation of states S_1_ and T_1_ in **Ir**_**2**_**(dppm)**_**3**_**(acac)**_**2**_ and **Ir**_**3**_**(dppm)**_**4**_**(acac)**_**3**_. With the help of TD-DFT calculations (see below), we substantiate that this is a consequence of the combined effect of the lowest excited states having strong charge transfer (d → π*) character and of their relatively extended delocalization within more metal centers and cyclometalated ligands, compared to mononuclear complexes such as **Ir(dppm)**_**2**_**(acac)**.^21^ Indeed, both charge transfer character and extended delocalization ought to decrease the exchange energy (K) of the electronic configuration and decrease the gap between the corresponding singlet and triplet states (2K). Hence, a relatively strong spectral overlap of T_1_ → S_0_ phosphorescence with S_1_ → S_0_ absorption band in **Ir**_**2**_**(dppm)**_**3**_**(acac)**_**2**_ and **Ir**_**3**_**(dppm)**_**4**_**(acac)**_**3**_ seems rational. It is noted that in frozen toluene glass at *T* = 77 K, the emission spectra of the two complexes are slightly hypsochromically shifted, which we rationalize to be due to the limited reorganization of the media affecting the stabilization of the emitting state. Also, at *T* = 77 K, vibrational shoulders of emission spectra become resolved at 638 nm for **Ir**_**2**_**(dppm)**_**3**_**(acac)**_**2**_ and at 644 nm for **Ir**_**3**_**(dppm)**_**4**_**(acac)**_**3**_ (Figure 1).

**Figure 2:**
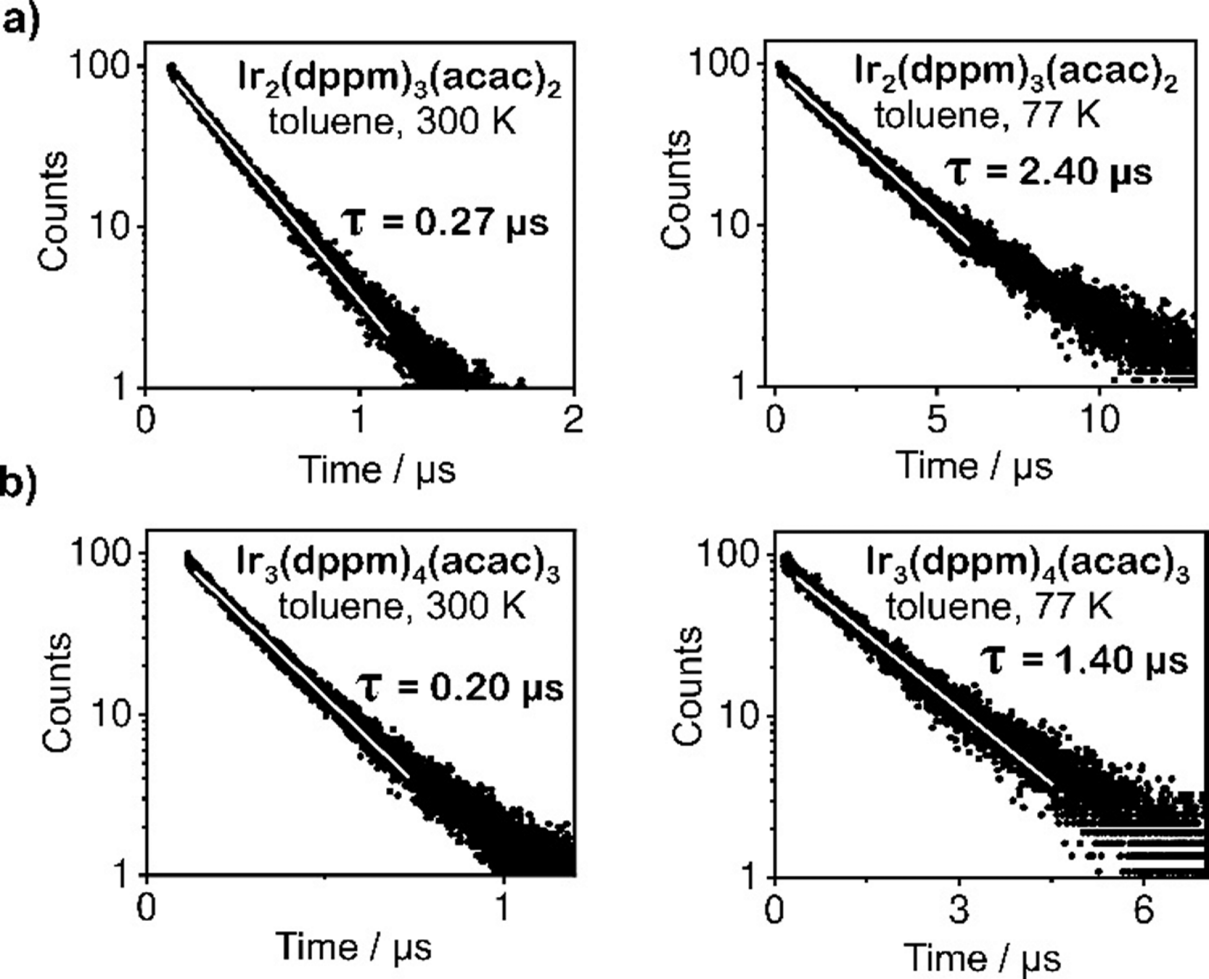
Emission decay traces measured for degassed toluene solution (*c* ≈ 10^–5^ M) of (a) **Ir**_**2**_**(dppm)**_**3**_**(acac)**_**2**_ and (b)**Ir**_**3**_**(dppm)**_**4**_**(acac)**_**3**_ at 300 K and at 77 K as indicated in the insets. The white line on the black dots of experimental data represents the best fit of the exponential decay function.

The emission decay time constants and quantum yields, measured for degassed toluene solutions (*c* ≈ 10^–5^ M) under ambient temperature, are τ = 0.27 μs and Φ_PL_ = 0.65 (65%) for **Ir**_**2**_**(dppm)**_**3**_**(acac)**_**2**_; and τ = 0.20 μs and Φ_PL_ = 0.60 for **Ir**_**3**_**(dppm)**_**4**_**(acac)**_**3**_. The traces of time-correlated single photon counting (TCSPC) measurements are shown in Figure 2. The corresponding radiative rate (*k*_r_ = Φ_PL_/τ) and radiative decay time constant (τ_r_ = τ/Φ_PL_ = 1/*k*_r_) values are *k*_r_ = 2.41 × 10^6^ s^–1^ and τ_r_ = 0.42 μs for dinuclear **Ir**_**2**_**(dppm)**_**3**_**(acac)**_**2**_; and *k*_r_ = 3.0 × 10^6^ s^–1^ and τ_r_ = 0.33 μs for trinuclear **Ir**_**3**_**(dppm)**_**4**_**(acac)**_**3**_.

The obtained radiative decay time value of the dinuclear **Ir**_**2**_**(dppm)**_**3**_**(acac)**_**2**_ is in the range of the fastest emitting dinuclear Ir(III) complexes reported so far and about two times shorter than that of the mononuclear **Ir(dppm)**_**2**_**(acac)** at the same conditions.^21^ Meanwhile, the radiative decay time of trinuclear **Ir**_**3**_**(dppm)**_**4**_**(acac)**_**3**_ of τ_r_ = 0.33 μs is unprecedentedly short for T_1_ → S_0_ phosphorescence, making the complex the fastest phosphorescing material known so far. At *T* = 77 K in frozen toluene glass, phosphorescence efficiency increases to unity for **Ir**_**2**_**(dppm)**_**3**_**(acac)**_**2**_ and reaches 0.9 (90%) for **Ir**_**3**_**(dppm)**_**4**_**(acac)**_**3**_ (Table 1), which is explained by the significantly reduced rate of nonradiative relaxation processes in rigid media at low temperatures. Interestingly, the radiative rates of the two complexes are also lower at *T* = 77 K compared to those under ambient conditions, though the decrease is not as significant as for nonradiative decay rates. This, in Ir(III) complexes, occurs due to a zero field splitting (ZFS) of the emitting T_1_ state, causing a relatively inefficient thermal population of the fastest emitting (highest energy) substate of T_1_ at lower temperatures. Another noteworthy finding is that in doped polystyrene films (*c* < 1 wt %), both complexes, **Ir**_**2**_**(dppm)**_**3**_**(acac)**_**2**_ and **Ir**_**3**_**(dppm)**_**4**_**(acac)**_**3**_, demonstrate the increase in the phosphorescence efficiency, as compared to the toluene solutions under ambient conditions (Table 1). This is associated with both increased radiative rates and reduced nonradiative relaxation rates in polystyrene films (Table 1). Indeed, a more rigid media of the film may suppress extensive geometry reorganizations of the molecule in the excited state and hence the nonradiative relaxation processes too. Also, the relatively high rigidity of the film may suppress the rotation of the noncoordinated terminal phenyl groups of **dppm** ligands in both complexes, keeping them more in plane with the rest of the ligand and improving the molecule’s chromophore properties. This results in a higher average oscillator strengths of the excited singlet states (*f*(S_n_ ↔ S_0_)) spin–orbit coupled with the phosphorescing T_1_ state, and hence in a faster T_1_ → S_0_ phosphorescence.^14^ A similar increase in the phosphorescence rate in PS film was also observed for mononuclear **Ir(dppm)**_**2**_**(acac)**.^21^ It is noted that in PS film under ambient temperature, the radiative decay time of phosphorescence of trinuclear **Ir**_**3**_**(dppm)**_**4**_**(acac)**_**3**_ is as short as only τ_r_ = 0.3 μs.

## Low Temperature Optical Spectroscopy

The rate of phosphorescence stemming from a triplet substate of a mixed ππ* (LC) and dπ* (MLCT) character in Ir(III) complexes is largely defined by the strength of spin–orbit coupling (SOC) of the triplet substate with the higher laying singlets and by the oscillator strengths of those singlets with respect to the ground state as approximated by the following equation:^15,38−40^
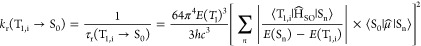
1herein T_1,i_ is the *i*-th substate of T_1_, *k*_r_(T_1,i_ → S_0_) is the radiative rate of the T_1,i_ → S_0_ transition, τ_r_(T_1,i_ → S_0_) is the corresponding radiative decay time, *h* and *c* are Planck’s constant and the speed of light, ⟨T_1_|Ĥ_SO_|S_n_⟩ is the SOC matrix element (SOCME), and ⟨S_0_|μ̂|S_n_⟩ is the transition dipole moment of S_n_ → S_0_ related to the oscillator strength of S_n_ as *f*(S_n_ ↔ S_0_) ∝ |⟨S_0_|μ̂|S_n_⟩|^2^, respectively. The experimental phosphorescence rate of the T_1_ state represents (assuming efficient equilibration between the three triplet substates (T_1,i_) a Boltzmann average of its three substates:^41,42^
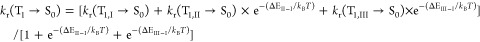
2Herein I, II, and III are the three substates (*i*-th) of the T_1_ state, according to ascending energy, ΔE_II–I_ and ΔE_III–I_ are energy separations between the T_1_ substates, *k*_B_ is the Boltzmann constant, and *T* is the absolute temperature, respectively. Δ*E*_III–I_ representing the energy difference between the lowest (I) and highest triplet substates is also denoted as zero-field splitting (ZFS). Thus, according to eq 2, the overall rate of T_1_ → S_0_ phosphorescence will vary with temperature that governs the population efficiency of the higher laying T_1_ substates. Assuming emission quantum yields of the complexes in this temperature range remain near unity, similar to that measured at *T* = 77 K, so that measured decay time constants are close to radiative decay time constants, eq 2 can be simplified to:
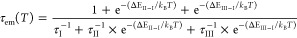
3which is now a convenient relation to use for analysis of measured decay time constants as the function of properties of T_1_’s substates and energy gaps between them. This opens the possibility for temperature-dependent investigations to reveal individual emission rates of T_1_ substates and ZFS value.^43,44^ Thus, the dilute toluene solution samples of **Ir**_**2**_**(dppm)**_**3**_**(acac)**_**2**_ and **Ir**_**3**_**(dppm)**_**4**_**(acac)**_**3**_ were investigated at cryogenic temperatures in the range 1.6 K ≤ T ≤ 120 K where toluene remains frozen under vacuum.

**Figure 3:**
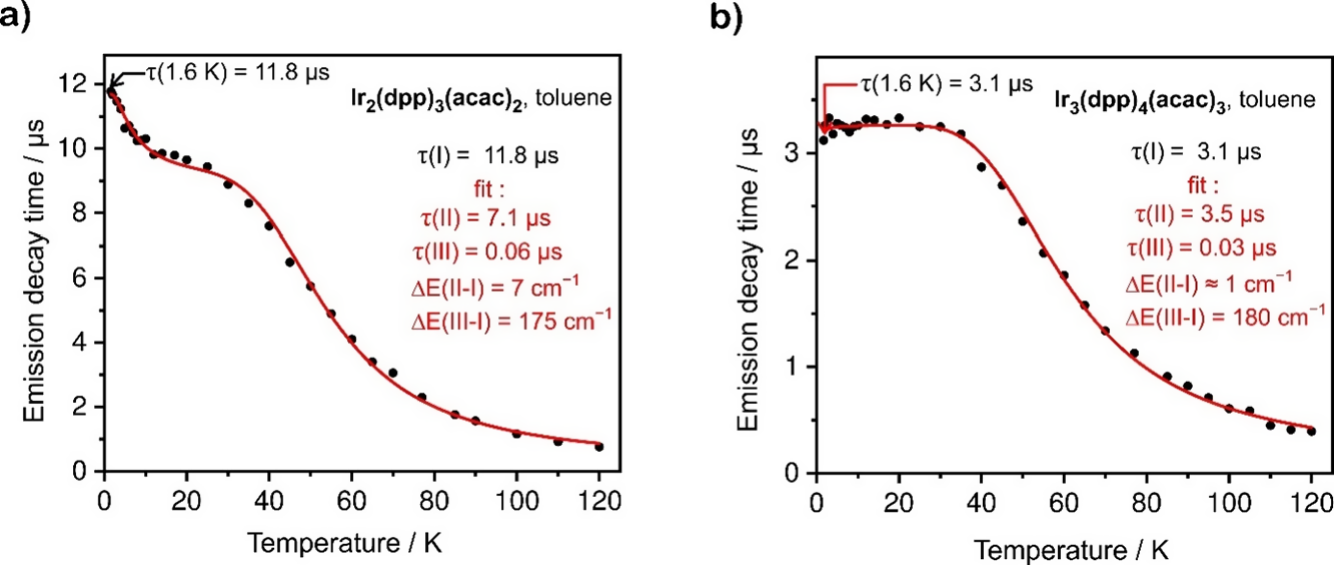
Emission decay times of (a) **Ir**_**2**_**(dppm)**_**3**_**(acac)**_**2**_ and (b) **Ir**_**3**_**(dppm)**_**4**_**(acac)**_**3**_ as a function of temperature (black dots), and the best fit of eq 3 to the experimental values (red line).

Figure 3a shows the measured emission decay times of **Ir**_**2**_**(dppm)**_**3**_**(acac)**_**2**_ plotted as a function of temperature. The decay time at 1.6 K is τ(1.6 K) = 11.8 μs, several times shorter than that of mononuclear **Ir(dppm)**_**2**_**(acac)** (τ(1.6 K) = 66 μs)^21^ measured under the same conditions. It is interpreted that emission at this temperature mainly stems from the lowest T_1_ substate (T_1,i_). With the increase in temperature, the emission decay time drops to reach a *quasi*-plateau at the 10–25 K temperature range where substrates I and II are thermally equilibrated with an average decay time value of about 9.7 μs. With a further increase in temperature, the emission decay time drops due to the thermal population of the fastest-emitting T_1_ substate III, and at *T* = 120 K reaches the value of 0.75 μs. Analysis of the experimental data using eq 3. with fixed τ(I) = τ(1.6 K) = 11.8 μs suggests the following parameter values for the best fit: τ(II) = 7.1 μs, τ(III) = 0.06 μs (60 ns), ΔE(II–I) = 7 cm^–1^ and ΔE(III–I) = 175 cm^–1^. According to these data, dinuclear **Ir**_**2**_**(dppm)**_**3**_**(acac)**_**2**_ has notably faster emitting T_1_ substates I and III, compared to those of mononuclear **Ir(dppm)**_**2**_**(acac)** (τ(I) = τ(1.6 K) = 66 μs, τ(II) = 7.3 μs, τ(III) = 0.19 μs, ΔE(II–I) = 14 cm^–1^ and ΔE(III–I) = 210 cm^–1^).^21^ A similar study of trinuclear **Ir**_**3**_**(dppm)**_**4**_**(acac)**_**3**_ is shown in Figure 3b. The two lowest substates of T_1_, I and II, are thermally equilibrated early at low temperatures due to a particularly small energy gap between them of about 1 cm^–1^. The plateau of the average emission decay time of the two lowest T_1_ substates continues up to *T* = 35 K. The average value of ca. 3.2 μs is about three times shorter than that in the dinuclear **Ir**_**2**_**(dppm)**_**3**_**(acac)**_**2**_, indicating a stronger average SOC mixing of the T_1_ substates with singlet states. An increase in temperature above 35 K is associated with the population of the highest T_1_ substate III and a significant drop in the emission decay time reaching 0.4 μs at *T* = 120 K. The fit of eq 3 suggests ZFS = 180 cm^–1^ and τ(III) = 0.03 μs (30 ns) for **Ir**_**3**_**(dppm)**_**4**_**(acac)**_**3**_ (Figure 3b).

Extrapolation of the thus obtained low-temperature data for the T_1_ state properties to the room temperature conditions, using *T* = 300 K in eq 3, suggests radiative phosphorescence decay time of τ_av_(300 K) = 0.32 μs for dinuclear **Ir**_**2**_**(dppm)**_**3**_**(acac)**_**2**_ and τ_av_(300 K) = 0.19 μs for trinuclear **Ir**_**3**_**(dppm)**_**4**_**(acac)**_**3**_. It is noted that the value of the trinuclear complex is corrected to account for the emission quantum yield in glassy toluene of 90% as measured at *T* = 77 K, e.g., the given value is after division by 0.9. These extrapolated values predict a bit faster T_1_ → S_0_ radiative rate than what is found experimentally at room temperature (Table 1). Such a deviation may arise from modification of the T_1_ state’s properties in liquid media at room temperature, as well as from the relative crudeness of the data obtained at low temperatures, for example, due to the possible nonuniformity of emission quantum yield in the temperature range of investigation. It is noted that in the case of mononuclear **Ir(dppm)**_**2**_**(acac),** the rate value extrapolated from cryogenic temperature data agreed very well with the experimental room temperature value.^21^ Importantly, from the data analysis above, it can be concluded that the photoluminescence of both materials, dinuclear and trinuclear, is completely governed by the T_1_ → S_0_ transition up to room temperature. Indeed, at the determined high T_1,i_ → S_0_ transition rates in these materials, there is little chance for emission through thermal activation of a state above T_1,III_, even at its close energetic proximity. Another noteworthy finding is that with more than a two-fold increase in the T_1_ → S_0_ phosphorescence rate from mononuclear **Ir(dppm)**_**2**_**(acac)** to dinuclear **Ir**_**2**_**(dppm)**_**3**_**(acac)**_**2**_, the analogous increase from mononuclear **Ir(dppm)**_**2**_**(acac)** to trinuclear **Ir**_**3**_**(dppm)**_**4**_**(acac)**_**3**_ is less than three-fold (Table 1). We attribute this to the different electronic properties of the central metal atom from the two peripheral metal atoms in the trinuclear complex, which may compromise the SOC efficiency of T_1_ with singlet states (vide infra).

Interestingly, the T_1_ ZFS value of 175 cm^–1^ in dinuclear **Ir**_**2**_**(dppm)**_**3**_**(acac)**_**2**_ and of 180 cm^–1^ in trinuclear **Ir**_**3**_**(dppm)**_**4**_**(acac)**_**3**_ are both slightly smaller than T_1_ ZFS of 210 cm^–1^ found for the mononuclear **Ir(dppm)**_**2**_**(acac)**.^21^ Such a decrease in the ZFS value in multinuclear complexes is unexpected and contrasts with the findings for other pairs of mononuclear and dinuclear complexes.^24,27^ In the transition metal complexes, the ZFS size is determined by different SOC strengths for different orientations of electron spins caused by the filed anisotropy at the SOC center(s) (metal ion(s)).^45^ Therefore, the decreased ZFS value in the dinuclear and trinuclear complexes is indicative of lowered overall field anisotropy at the metal centers. In the mononuclear **Ir(dppm)**_**2**_**(acac)**, two dppm ligands are aligned (Chart 1), giving the molecule and orbitals involved in SOC of the T_1_ state a shape elongated in one direction (along an arbitrary *z*-axis), which works for a relatively strong field anisotropy at the SOC center (gives a relatively large ZFS parameter D).^21^ In the dinuclear **Ir**_**2**_**(dppm)**_**3**_**(acac)**_**2**_ and trinuclear **Ir**_**3**_**(dppm)**_**4**_**(acac)**_**3**_, however, the alignment of the two dppm ligands at different metal centers cover different spatial directions (close to mutually perpendicular) so that the molecules and the orbitals appear less elongated along one axis. This, we conclude, decreases the field anisotropy (a smaller ZFS parameter D) “seen” by the spin–orbit coupled electron. Indeed, the above-referenced dinuclear complexes, with T_1_ ZFS values larger than that in their mononuclear analogues,^24,27^ do not feature such mutually compensating alignment of the ligands at the two metal centers. It is also noteworthy that the decrease of the ZFS parameter D of the T_1_ state with the expansion of electron delocalization to a new dimension was reported for π-stacked dimers of porphyrin complexes in comparison to their monomers.^46,47^ This structural feature of **Ir**_**2**_**(dppm)**_**3**_**(acac)**_**2**_ and **Ir**_**3**_**(dppm)**_**4**_**(acac)**_**3**_, affording relatively small T_1_ ZFS, should improve the thermal population of the higher laying and faster emitting T_1_ substates and thus contribute to the enhancement of phosphorescence rate under ambient temperature.

## DFT Calculations and Theory

Electronic structures of **Ir**_**2**_**(dppm)**_**3**_**(acac)**_**2**_ and **Ir**_**3**_**(dppm)**_**4**_**(acac)**_**3**_ were computed utilizing the density functional theory (DFT) approach at the M11L^48,49^/def2-SVP^50^ level with effective core potentials (ECP) for iridium atoms and using Gaussian 09^51^ code. Vertical excitations were computed with the time-dependent DFT (TD-DFT) approach. The C-PCM polarizable continuum model^52^ with parameters of toluene was applied to all the calculations to account for the solvation effect. The ground state optimized ΛΛ-isomer of **Ir**_**2**_**(dppm)**_**3**_**(acac)**_**2**_ was calculated 11.16 kcal/mol more stable than the ΛΔ-isomer, which is due to the steric hindrances in the latter, as mentioned above. For calculation of excited states and electronic structure analysis, we therefore used ΛΛ-isomer of **Ir**_**2**_**(dppm)**_**3**_**(acac)**_**2**_ and ΛΛΛ-isomer of **Ir**_**3**_**(dppm)**_**4**_**(acac)**_**3**_. The structures were optimized for both ground state (S_0_) and the lowest excited triplet state (T_1_) electronic configurations. The corresponding atomic Cartesian coordinates are given in the Supporting Information. Unless stated otherwise, the data discussed below refer to the T_1_ state optimized geometries as more relevant to the photoluminescent properties of these complexes.

**Figure 4:**
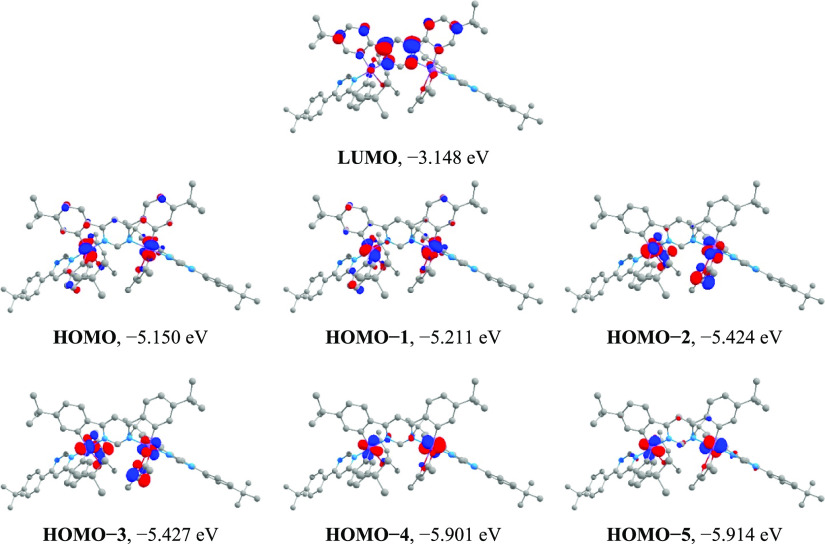
Iso-surface (iso-value = 0.05) contour plots of selected molecular orbitals of **Ir**_**2**_**(dppm)**_**3**_**(acac)**_**2**_.

According to TD-DFT calculations, the state T_1_ of dinuclear **Ir**_**2**_**(dppm)**_**3**_**(acac)**_**2**_ has HOMO → LUMO (98%) orbital parentage (Table S5). The HOMO is localized over the two metals evenly with 44% of net contribution; on the doubly cyclometalated bridging **dppm** ligand (*dppm2*) with 24% contribution; over two terminal **dppm** ligands (*dppm1* and *dppm3*) evenly with 22% of net contribution; and over two ancillary **acac** ligands evenly with 8% of net contribution (Table S3). The LUMO is almost entirely localized on the bridging **dppm** (*dppm2* in Chart 1) ligand, predominantly on the pyrimidine ring, with 90% contribution (Table S3). Contour plots of the MOs are shown in Figure 4. Hence, the T_1_ state of **Ir**_**2**_**(dppm)**_**3**_**(acac)**_**2**_ has strong metal-to-ligand charge transfer (^3^MLCT, ^3^dπ*) character contribution mixed with ligand-centered (^3^LC) character and, to a lesser extent, ligand-to-ligand charge transfer (LL′CT) character contributions. An unpaired electron in such a state, partially localized on the d-orbital of iridium, has a large chance of occurring at the Ir center with a strong SOC effect. Then, through its ^3^d_k_π* character, the T_1_ state can directly spin–orbit couple with a singlet state (S_n_) bearing ^1^*d*_*k*′_π*′ character. The corresponding SOC matrix element in eq 1 can be approximated as follows:
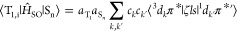
4Here, *c_k_* and *c*_*k*′_ are the normalized contributions of the *d_k_* and *d*_*k*′_ atomic orbitals to the molecular orbitals involved to direct excitations forming T_1_ and S_n_ states, respectively; *a*_T1_ and *a*_Sn_ are normalized configuration interaction (CI) coefficients of orbital transitions contributing to S_0_ → T_1_ and S_0_ → S_n_ excitations, respectively; ζ is the SOC constant of the center(s) associated with the d-orbitals, *l* is the angular momentum operator, and *s* is the spin momentum operator. To demonstrate, the HOMO → LUMO orbital transition contribution to the T_1_ state (S_0_ → T_1_ excitation) of **Ir**_**2**_**(dppm)**_**3**_**(acac)**_**2**_ has the corresponding configuration interaction coefficient of *a*_*T1*_ = 0.98 (98%), and metal contribution to the HOMO has the corresponding coefficient of *c*_*k*_ = 0.44 (44%) (Tables S3 and S5). Coefficients *a*_T1_ and *a*_Sn_ with coefficients *c_k_* and *c*_*k*′_ are to account for the probability of the electron occurring at the same heavy atom in both states. This is because the SOC effect, proportional to the fourth power of the nucleus’s charge (Z^4^) and to the inverse of the third power of distance to the nucleus (1/*r*^3^), is strong only close to a heavy atom nucleus.^18,53^ Therefore, a spin–orbit coupled electron can “mix” only those state wave functions, which are contributed by the same SOC-providing center(s). This, basically, is a requirement that *d_k_* and *d*_*k*′_ belong the same atom(s) and that π* = π*′ as an electron on a π* orbital is not spin–orbit coupled to an appreciable extent and must remain the same for the two states. Also, to conserve the total momentum (spin+orbital) of the spin–orbit coupled electron upon spin-flip, it is required that *d_k_* ≠ *d*_*k*′_ (also known as El-Sayed’s rule^54^). Then, the flip of spin vector of the spin–orbit coupled electron between the two states can be compensated by the change of angular momentum vector to keep the total momentum vector conserved. Hence, SOC matrix elements in eq 4 will be appreciable only for pairs of states fulfilling both *d_k_* ≠ *d*_*k*′_, with *d_k_* and *d*_*k*′_ belonging to the same SOC center(s), and π* = π*′, and will vanish for other pairs of states as mathematically ensured by the spin and angular momentum operators and matrix multiplication rules. It is noted that although El-Sayed’s rule is only a “thumb rule” and may miss some details, it still is important for the general trend of SOC efficiency between the states depending on their electronic properties and can be a reliable guide for qualitative and semi-quantitative analyses such as presented in this contribution.

Equation 4 shows that the higher the MLCT (dπ*) character extent of a given state, the larger the SOC matrix elements with other states it can have. Then, from the requirements *d_k_* ≠ *d*_*k*′_ and π* = π*′, it follows that the T_1_ state (1.95 eV, 98% HOMO → LUMO) can have a large SOC matrix element with singlet states of HOMO–*n* → LUMO orbital parentage, where HOMO–*n* has a significant contribution by a metal atomic orbital (*d*_*k*′_) with angular momentum orientation different from that of d_k_ atomic orbital contributing to the HOMO. In Ir(III) complexes, *d_k_* and *d*_*k*′_ orbitals are represented by the three *quasi*-degenerate t_2g_ symmetry 5d-orbitals (*d_xy_*, *d_xz_*, *d_yz_*). In dinuclear complexes of *C*_*2*_ symmetry, electronic coupling of the two coordination sites, symmetric and antisymmetric to C_2_ rotation, doubles the number of the t_2g_ 5d-orbital contributed MOs, where each MO is evenly contributed by both metals. In other words, instead of three MOs contributed by the t_2g_ atomic orbitals of one metal atom and another three MOs contributed by the t_2g_ atomic orbitals of the other metal atom, like in two independent mononuclear molecules, the same net metal contribution goes to six MOs each evenly contributed by the t_2g_ orbitals of both metal atoms (Figure 4). Accordingly, in dinuclear **Ir**_**2**_**(dppm)**_**3**_**(acac)**_**2**_, the T_1_ state bearing ^3^*d_k_*π* character from both metals can spin–orbit couple with four ^1^*d*_*k*′_π*′ character singlet states also contributed by both metal centers, with individual SOC matrix elements comparable to those in the analogous mononuclear complex. The analogous mononuclear Ir(III) complex, however, will have only three occupied MOs with significant t_2g_ atomic orbital contribution and hence a triplet state contributed by one of those MOs will have only two singlet states that can be directly spin–orbit coupled with.^16,21^ Hence, the number of singlet states that the T_1_ state can borrow oscillator strength from via SOC in a symmetric dinuclear complex is also doubled, compared to its mononuclear analogue, whereas the SOC matrix elements of each contributing path remains similar to that in the mononuclear complex.^24,27^ From the TD-DFT data we obtained, such singlet states in the dinuclear **Ir**_**2**_**(dppm)**_**3**_**(acac)**_**2**_ are S_7_ (2.30 eV, 98% HOMO–3 → LUMO), S_8_ (2.35 eV, 98% HOMO–2 → LUMO), S_16_ (2.80 eV, 54% HOMO–4 → LUMO), and S_17_ (2.81 eV, 79% HOMO–5 → LUMO) (Table S5). Note that state S_2_ of HOMO–1 → LUMO orbital parentage does not fulfill the *d_k_* ≠ *d*_*k*′_ requirement for SOC with the T_1_ state as HOMO and HOMO–1 are contributed by the same d-orbitals of the iridium atoms and differ only by symmetry to C_2_ symmetry operations. The doubled number of singlet states spin–orbit coupled to T_1_, compared to the mononuclear analogue, gives a larger sum of the SOC matrix elements in eq 1 and, consequently, a strong enhancement of the phosphorescence rate in the dinuclear complex. This agrees well with the experimental data for **Ir(dppm)**_**2**_**(acac)**(21) and **Ir**_**2**_**(dppm)**_**3**_**(acac)**_**2**_ (Table 1).

**Figure 5:**
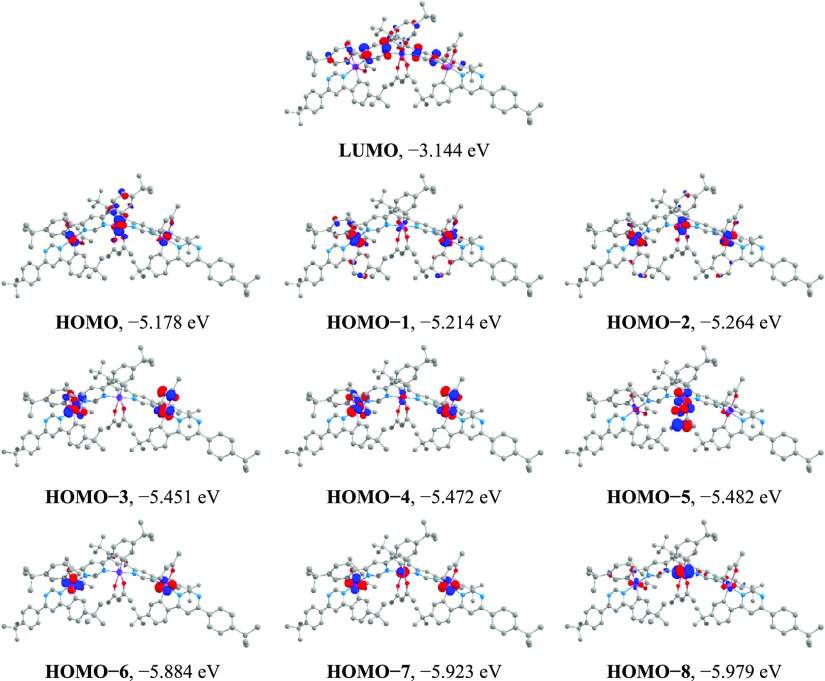
Iso-surface (iso-value = 0.05) contour plots of selected molecular orbitals of **Ir**_**3**_**(dppm)**_**4**_**(acac)**_**3**_.

The case of trinuclear **Ir**_**3**_**(dppm)**_**4**_**(acac)**_**3**_ is rather interesting. On the one hand, one could expect that trinuclear design would triple the SOC paths of the T_1_ state, compared to the mononuclear case, and enhance the phosphorescence rate yet further. On the other hand, the molecule has *C*_2_ symmetry, with the *C*_2_ axis going through the central Ir atom (*Ir2* on Chart 1) and bisecting the acac ligand coordinated to it, so that the two terminal iridium atoms are electronically identical to each other and different from the central one. Indeed, as the calculations show, the nine highest occupied MOs of **Ir**_**3**_**(dppm)**_**4**_**(acac)**_**3**_, from HOMO to HOMO−8, comprise even t_2g_ contribution from the two terminal iridium atoms, electronically coupled symmetric and antisymmetric to C_2_ rotation, and a different amount of t_2g_ contribution from the central iridium atom (Figure 5 and Table S4). According to the TD-DFT data, the T_1_ state (1.98 eV) of **Ir**_**3**_**(dppm)**_**4**_**(acac)**_**3**_ is of HOMO → LUMO (95%) orbital parentage (Table S6). The HOMO is localized on the two terminal iridium atoms evenly with a net contribution of 20%; on the central metal atom with a contribution of 24%; on the two doubly cyclometalated **dppm** ligands (*dppm2* and *dppm3* on Chart 1) evenly with a net contribution of 40%; on the two singly cyclometalated **dppm** ligands evenly with a net contribution of 8%; and on the three **acac** ligands with a net contribution of 8% (Figure 5 and Table S4). The LUMO is localized on the two doubly cyclometalated **dppm** ligands (*dppm2* and *dppm3* on Chart 1) evenly with a net contribution of 92%. Hence, the T_1_ state of **Ir**_**3**_**(dppm)**_**4**_**(acac)**_**3**_ is, as expected, of mixed ^3^MLCT/LC character. The total metal contribution to the HOMO of 44%, and hence, the MLCT extent of the T_1_ state is similar to that in mononuclear **Ir(dppm)**_**2**_**(acac)**(21) and dinuclear **Ir**_**2**_**(dppm)**_**3**_**(acac)**_**2**_. Importantly, according to the composition of the HOMO shown above, the contribution of the central iridium atom to the MLCT character of T_1_ state outweighs the net contribution of the two terminal Ir atoms. Hence, the central iridium atom will be the main SOC center for the T_1_ state. As such, for the correct description of spin–orbit coupling matrix elements, eq 4 should be modified to split the part of the terminal iridium atoms from the part of the central iridium atom.
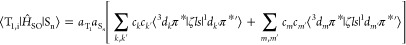
5On the right side of eq 5 , the first term in the bracket accounts for the terminal iridium atoms. Here *d_k_* and *d*_*k*′_ are atomic orbitals of the terminal Iridium atoms contributing to the MLCT character of the T_1_ and S_n_ states with weighs *c_k_* and *c*_*k*′_, respectively. The second member accounts for the part of the central Iridium atom where *d_m_* and *d*_*m*′_ are orbitals of the central iridium atom with *c_m_* and *c*_*m*′_ contribution weighs to the MLCT character of the T_1_ state and S_n_ state, respectively. Coefficients *a*_*T*_1__*a*_*S*_n__ represent the same as in eq 4.

Similar to the dinuclear case, in trinuclear **Ir**_**3**_**(dppm)**_**4**_**(acac)**_**3**_, a singlet state directly spin–orbit coupled with the T_1_ state should have HOMO–*n* → LUMO character where HOMO–*n* is contributed by the same Ir center(s) as HOMO but with different t_2g_ 5d-orbital(s) (*d_k_* ≠ *d*_*k*′_ and/or *d_m_* ≠ *d*_*m*′_). Accordingly, HOMO–*n* can be HOMO–3 through HOMO–8 (Figure 5). HOMO–1 has only a minor contribution of the central iridium atom fulfilling *d_m_* ≠ *d*_*m*′_. The corresponding singlet states are S_11_ (2.32 eV, 82% HOMO–3 → LUMO), S_14_ (2.34 eV, 85% HOMO–4 → LUMO), S_15_ (2.36 eV, 74% HOMO–5 → LUMO), S_30_ (2.76 eV, 82% HOMO–6 → LUMO), S_32_ (2.78 eV, 23% HOMO–7 → LUMO), S_33_ (2.80 eV, 39% HOMO–7 → LUMO), S_38_ (2.87 eV, 39% HOMO–8 → LUMO), and S_40_ (2.88 eV, 32% HOMO–8 → LUMO). All these states are within a 1 eV gap to the T_1_ state (1.98 eV, 95% HOMO → LUMO). However, due to the differences in MLCT character distribution between the central metal atom and the two terminal metal atoms in the two states, neither of these SOC paths exploits the MLCT character of the T_1_ state and of the corresponding singlet state to the full scale. For instance, HOMO–3 has an orbital node at the central Iridium atom (Figure 5) and is not contributed by it. Consequently, SOC of state S_11_ with the T_1_ state is limited to the terminal iridium atoms, meaning that less than half of the original MLCT character of the T_1_ state will contribute to this SOC path. In other words, the term accounting for the SOC part at the central iridium atom in eq 5 vanishes due to *c*_*m*′_ = 0. Thus, although, trinuclear **Ir**_**3**_**(dppm)**_**4**_**(acac)**_**3**_ has more SOC paths of T_1_ state with singlets, the SOC matrix element of each path is compromised, compared to those in mononuclear **Ir(dppm)**_**2**_**(acac)**(21) and dinuclear **Ir**_**2**_**(dppm)**_**3**_**(acac)**_**2**_. We rationalize that this is manifested by the relatively moderate enhancement of T_1_ → S_0_ phosphorescence rate from dinuclear **Ir**_**2**_**(dppm)**_**3**_**(acac)**_**2**_ to trinuclear **Ir**_**3**_**(dppm)**_**4**_**(acac)**_**3**_.

## Concluding Remarks

Complexes **Ir**_**2**_**(dppm)_3_(acac)**_2_ and **Ir**_**3**_**(dppm)**_**4**_**(acac)**_**3**_ are found to exhibit intense phosphorescence with extraordinarily high rates (*k*_r_ = Φ_PL_/τ). The corresponding radiative decay times (τ_r_ = 1/*k*_r_ = τ/Φ_PL_) are only τ_r_ = 0.36 μs for dinuclear **Ir**_**2**_**(dppm)**_**3**_**(acac)**_**2**_ and τ_r_ = 0.30 μs for trinuclear **Ir**_**3**_**(dppm)**_**4**_**(acac)**_**3**_, as measured in doped neat polystyrene film at room temperature. These values are unprecedented and set a new milestone for the phosphorescence rates of transition metal complexes. In agreement with these rate values, the cryogenic temperature investigations revealed a drastic increase in the emission rates of the individual T_1_ substates in the dinuclear **Ir**_**2**_**(dppm)**_**3**_**(acac)**_**2**_ and, especially, in trinuclear **Ir**_**3**_**(dppm)**_**4**_**(acac)**_**3**_, as compared to the mononuclear **Ir(dppm)**_**2**_**(acac)**. Evidently, such a high rate of phosphorescence in **Ir**_**2**_**(dppm)**_**3**_**(acac)**_**2**_ and **Ir**_**3**_**(dppm)**_**4**_**(acac)**_**3**_ is brought by the multinuclear design of these materials. Based on the electronic structure analysis, we interpret that multinuclear structure can afford a larger number of excited singlet states spin–orbit coupled with T_1_ state and lending oscillator strength to T_1_ → S_0_ transition. In dinuclear **Ir**_**2**_**(dppm)**_**3**_**(acac)**_**2**_, for instance, the sum of SOC matrix elements between T_1_ state and singlet states is roughly doubled (multiplied by the number of SOC centers (“nuclei”)), as compared to the mononuclear analogue. The effect of multinuclearity on the phosphorescence rate can be less prominent if the SOC centers of the molecule differ. This is because the matrix elements of each SOC path in such case is reduced, as found for **Ir**_**3**_**(dppm)**_**4**_**(acac)**_**3**_ where the central iridium atom is slightly different from the two terminal iridium atoms. In the case of **Ir**_**3**_**(dppm)**_**4**_**(acac)**_**3,**_ however, the larger overall number of SOC paths seems to take over the relatively reduced efficiency of each path and its phosphorescence rate still appears to be higher than that of dinuclear **Ir**_**2**_**(dppm)**_**3**_**(acac)**_**2**_. Nevertheless, to exploit the multinuclear design for enhancement of phosphorescence rate to the full extent, the molecular symmetry must ensure that all the SOC centers (“nuclei”) are electronically equal and coupled, similarly to the case of dinuclear **Ir**_**2**_**(dppm)**_**3**_**(acac)**_**2**_. Also, one can anticipate that the SOC centers in the molecule better be rigidly bridged to prevent symmetry-breaking distortions in the T_1_ state, which may lift the electronic equality of the SOC centers.

The smaller T_1_ state ZFS of both **Ir**_**2**_**(dppm)**_**3**_**(acac)**_**2**_ (175 cm^–1^) and **Ir**_**3**_**(dppm)**_**4**_**(acac)**_**3**_, (180 cm^–1^) compared mononuclear **Ir(dppm)**_**2**_**(acac)** (210 cm^–1^)^21^ is another finding that knows no precedence. We rationalize that MOs involved in SOC of state T_1_ in **Ir**_**2**_**(dppm)**_**3**_**(acac)**_**2**_ and **Ir**_**3**_**(dppm)**_**4**_**(acac)**_**3**_ appear less elongated in one direction due to the alignment of the coordinated ligands in different directions at different coordination centers. Hence, the overall field anisotropy, seen by the spin–orbit coupled electron, is reduced (smaller ZFS parameter D), resulting in a smaller ZFS value. This structural effect may also be of use in designing fast phosphors as a smaller ZFS means more efficient thermal activation of the higher laying and faster emitting T_1_ substates.

## Supporting Information Available

Experimental information; DFT geometries; DFT and TD-DFT output data; and NMR characterization, mass spectrometry, and elemental analysis data (PDF)
