# Supplementary material for: Trinuclear Cyclometalated Iridium(III) Complex Exhibiting Intense Phosphorescence of an Unprecedented Rate
Source: Inorg Chem. 2023 Dec 28;63(2):1317–27. doi: 10.1021/acs.inorgchem.3c03810 (PMC10792602; doi:10.1021/acs.inorgchem.3c03810)
Supplement: Supplementary file 1 — ic3c03810_si_001.pdf [file ic3c03810_si_001.pdf]

Supporting information

Trinuclear Cyclometalated Iridium(III) Complex Exhibiting Intense Phosphorescence of an Unprecedented Rate

Marsel Z. Shafikov[a]*, Andrey V. Zaytsev[b], Valery N. Kozhevnikov[b]*

[a] *Institut für Physikalische und Theoretische Chemie, Universität Regensburg, Universitätsstrasse 31, Regensburg, D-93053, Germany.*

[b] *Department of Applied Sciences, Northumbria University, Newcastle upon Tyne, NE1 8ST, U.K.*

* E-mails:
shafikoff@gmail.com (M.Z.S.)
valery.kozhevnikov@northumbria.ac.uk (V.N.K.)

Synthesis and chemical characterization.

General

All solvents and reagents were purchased from commercial suppliers and used without further purification. NMR spectra were recorded on a JEOL ECS400FT Delta spectrometer (399.78 MHz for 1H NMR). Chemical shifts are reported in parts per million (ppm) relative to a tetramethylsilane internal standard. Elemental analysis was carried out on ELEMENTAR vario MICRO CUBE instrument at central analytical services of the University of Regensburg. Mass-spectroscopy (FD-MS) was performed on a JEOL AccuTOF GCX instrument at the central analytical services of the University of Regensburg.

Synthesis

Ligand

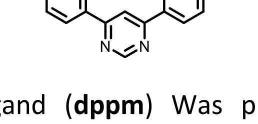

4,6-di(4-tert-butylphenyl)pyrimidine ligand (**dppm**) Was prepared according to the procedure reported earlier.[1]

Complexes

4,6-di(4-tert-butylphenyl)pyrimidine (1033 mg, 3 mmol) was dissolved in 2-ethoxyethanol (45 mL) at room temperature. $IrCl_3.nH_2O$ (705 mg, 2 mmol) was added at room temperature. The reaction flask

was submerged into oil bath preheated to 110°C. The mixture was stirred at 110°C for 3 days under argon atmosphere. Sodium acetylacetonate (701 mg, 5 mmol) and ethanol (17 mL) were added and the mixture was heated under reflux for 4 h. The mixture was allowed to cool to room temperature and filtered. The collected precipitate of the mononuclear **Ir(dppm)$_2$(acac)** complex was washed with ethanol (3 × 5 mL). All ethanolic filtrates were combined and evaporated to dryness. Water (20 mL) was added to the residue and the mixture was sonicated for 5 min. The precipitate was filtered, washed with water and air-dried. To eliminate the rest of the mononuclear complex, the product was subjected to column chromatography on silicagel eluting with DCM and collecting the dark-red fraction. Solvent was evaporated to give a dark-red solid (103 mg), which was further purified by preparative TLC (Figure S1) using pet.ether:AcOEt:DCM (8:1:1) as an eluent to give dinuclear **Ir$_2$(dppm)$_3$(acac)$_2$** (36 mg, 2.2%) and trinuclear **Ir$_3$(dppm)$_4$(acac)$_3$** (16 mg, 1.1 %) as dark red solids.

Ir$_2$(dppm)$_3$(acac)$_2$ (ΛΛ /ΔΔ)

^{1}H NMR (400 MHz, CDCl$_3$) δ 1.07 (18 H, s), 1.12 (18 H, s), 1.41 (18 H, s), 1.69 (6 H, s), 1.91 (6 H, s), 5.18 (2 H, s), 6.37 (2 H, d, *J* = 2.0 Hz), 6.57 (2 H, d, *J* = 1.8 Hz), 6.98 (4 H, 2 × dd), 7.62 (4 H, app. d), 7.67 (2 H, d, *J* = 8.3 Hz), 7.72 (2 H, d, *J* = 8.3 Hz), 8.08 (2 H, s), 8.12 (1 H, s), 8.17 (4 H, app. d), 9.09 (2 H, s), 9.14 (1 H, s).

^{13}C NMR (101 MHz, CDCl$_3$)
δ 29.0 (CH$_3$), 29.3 (CH$_3$), 31.2 ((CH$_3$)$_3$), 31.4 ((CH$_3$)$_3$), 34.7 (quat.), 35.1 (quat.), 101.4 (CH),104.0 (CH), 108.1 (quat.), 118.9(CH), 119.0 (CH), 125.4 (CH), 125.9 (CH), 126.2 (CH), 127.4 (CH), 130.1 (CH), 130 .2 (CH), 133.4 (quat.), 139.3 (quat.), 139.8 (quat.), 151.6 (quat.), 152.2 (quat.), 154.1 (quat.), 154.6 (quat.), 155.3 (quat.), 156.1 (quat.), 157.8 (CH), 163.7 (quat.), 174.8 (quat.), 175.2 (quat.), 185.0 (qu at.), 185.9 (quat.).

Field desorption mass-spectrometry (FD-MS): simulated for [M]$^+$ ($C_{82}H_{94}Ir_2N_6O_4$) 1612.6608, found 1612.6208.

Elemental analysis. Calculated for $C_{82}H_{94}Ir_2N_6O_4$: C 61.09, H 5.88, N 5.21 %; found C 60.50, H 6.12, N 4.77 %.

Ir$_3$(dppm)$_4$(acac)$_3$ (ΛΛΛ/ΔΔΔ)

^{1}H NMR (400 MHz, CDCl$_3$) δ 1.06 (18 H, s), 1.08 (18 H, s), 1.13 (18 H, s),1.41 (18 H, s), 1.67 (6 H, s), 1.79 (6 H, s), 1.91 (6 H, s), 5.09 (1 H, s), 5.17 (2 H, s), 6.34 (2 H, d, *J* = 1.9 Hz), 6.50 (2 H, d, *J* = 1.8 Hz), 6.56 (2 H, d, *J* = 1.8 Hz), 6.94 (2 H, dd, *J* = 8.2 and 1.9 Hz), 6.99 (2 H, dd, *J* = 8.3 and 1.9 Hz), 7.03 (2 H, dd, *J* = 8.3 and 1.9 Hz), 7.62 (4 H, app. d), 7.65 (2 H, app.d), 7.73 (4 H, app dd), 8.08 (2 H, s), 8.12 (2 H, s), 8.16 – 8.18 (4 H, app. d), 9.06 (2 H, d, *J* = 1.8 Hz), 9.11 (2 H, s).

Field desorption mass-spectrometry (FD-MS): simulated for [M]$^+$ ($C_{111}H_{127}Ir_3N_8O_6$) 2245.8766, found 2245.7269.

Elemental analysis. Calculated for $C_{111}H_{127}Ir_3N_8O_6$: C 59.36, H 5.70, N 4.99 %; found C 59.43, H 5.95, N 4.56 %.

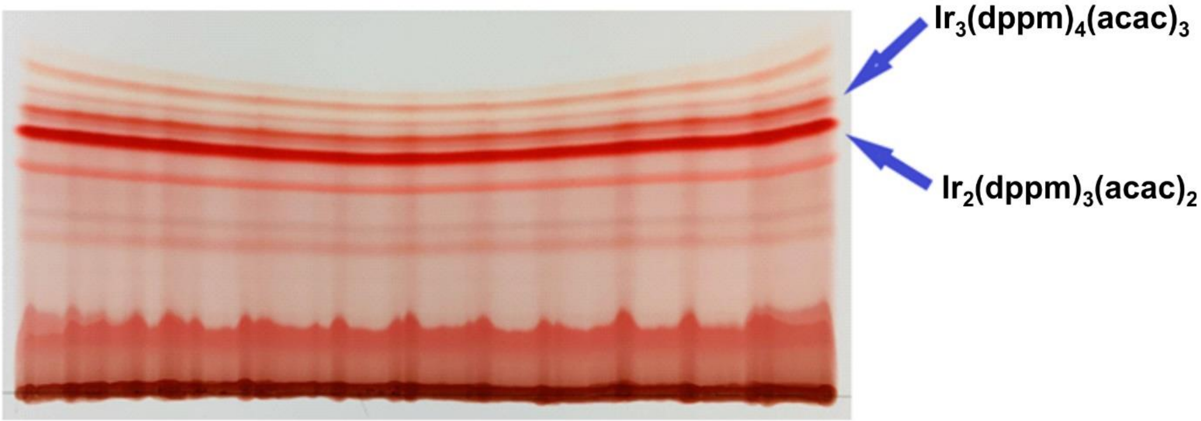

Figure S1. A picture of the preparative TLC.

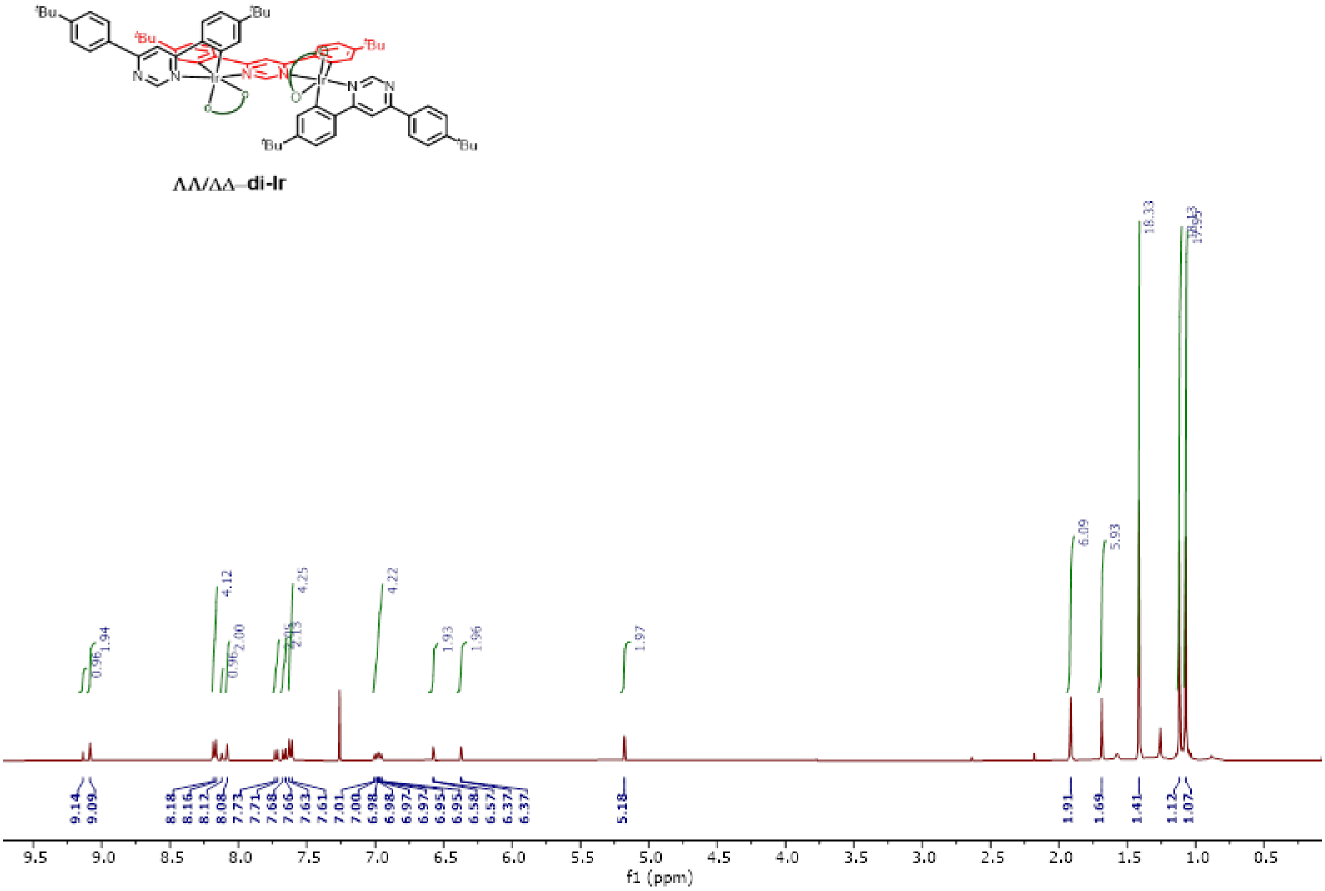

Figure S2. ^{1}H NMR spectrum of **$Ir_2(dppm)_3(acac)_2$** in chloroform-D.

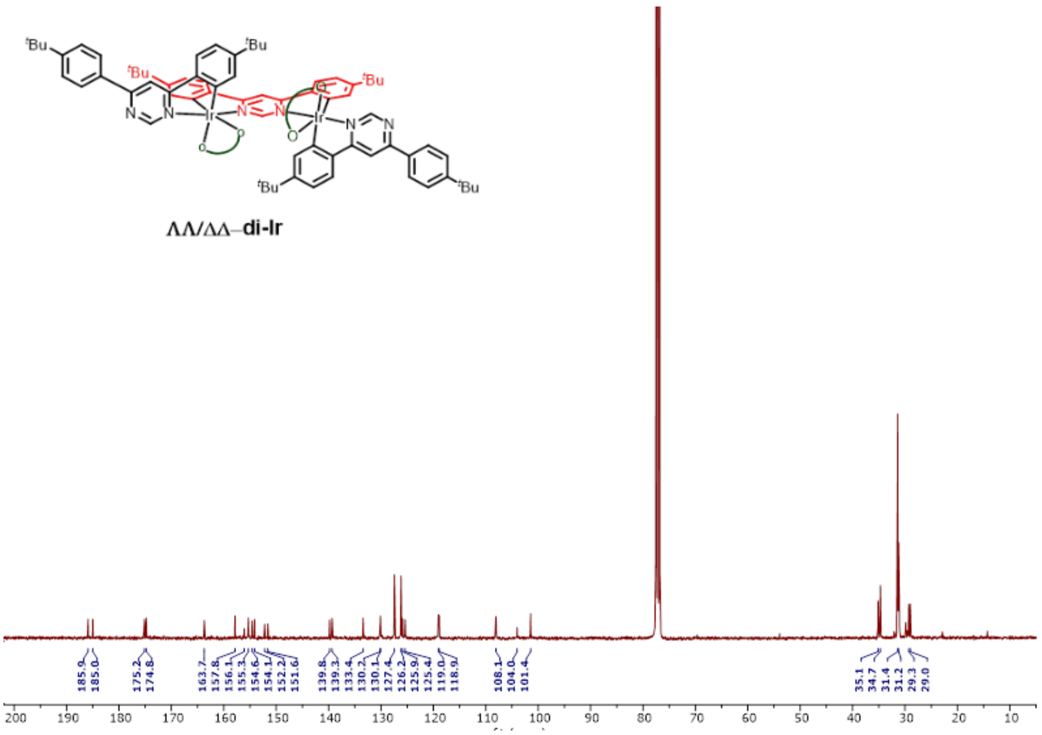

Figure S3. $^{13}C\{^1H\}$ NMR spectrum of **$Ir_2(dppm)_3(acac)_2$** in chloroform-D.

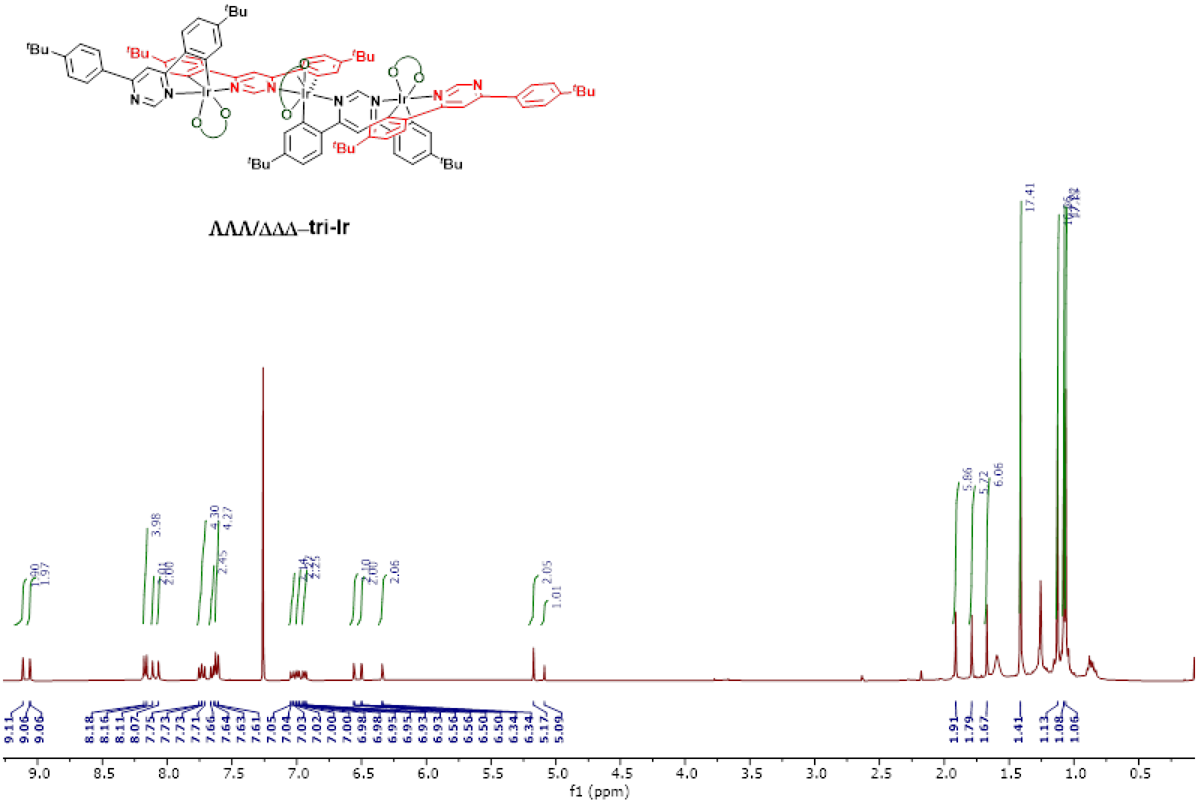

Figure S4. ^1H NMR spectrum of **$Ir_3(dppm)_4(acac)_3$** in chloroform-D.

Optical spectroscopy. The steady state photophysical measurements were performed on solutions of the complexes in spectroscopic grade toluene ($c \approx 10^{-5}$ M) and as doped in neat polystyrene film ($c \ll 1$ wt. %). The UV-Vis absorption spectra were measured with a Varian Cary 300 double beam spectrometer. The emission and excitation spectra were measured with a Horiba Jobin Yvon Fluorolog-3 steady-state fluorescence spectrometer. The emission decay times were measured with a PicoBright PB-375 pulsed diode laser (λ_{exc} = 378 nm, pulse width 100 ps) used as the excitation source, and the PL signal was detected with a cooled photomultiplier attached to a FAST ComTec multichannel scalar PCI card with a time resolution of 250 ps. The PL quantum yields were determined with a Hamamatsu C9920-02 system equipped with a Spectralon® integrating sphere. The low temperature photoluminescent properties were investigated with the sample deposited in a helium cryostat (Cryovac Konti Cryostat IT) where the helium gas flow, gas pressure, and heating were controlled. Thus, the temperature in the cryostat could be varied between 1.6 and 130 K. Room temperature quantum yield and decay time measurements of solutions were measured for the same samples degassed by freeze-pump-thaw procedure, repeated 4 times with pressure of vacuum reaching 10^{-5} mbar, and sealed with a Teflon valve. The sealed samples reproduced the measurement data next day that testify the quality of sealing. The quantum yield and decay times of doped PS films were measured under nitrogen atmosphere.

Computations. All calculations were carried out with the Gaussian 09 package[2] utilizing the DFT approach with the M11L functional[3] and the def2-SVP basis set[4] including ECPs for the Ir(III) ion. Geometry optimizations were conducted with "tight" criteria. The C-PCM solvation model[5] applied with solvent parameters for toluene.

Table S1. The coordination bond lengths (in angstrom) in DFT optimized ground state (S_0) and T_1 state geometries of **$Ir_2(dpp)_3(acac)_2$**.

**Parameter**	**Ground state (S_0)**	**state T_1**
Ir1−C_dpp1	1.987	1.981
Ir1−N_dpp1	2.029	2.035
Ir1−C_dpp2	1.990	1.982
Ir1−N_dpp2	2.024	2.023
Ir1-O1 (trans to C_dpp1)	2.120	2.113
Ir1-O2	2.127	2.125
Ir2−C_dpp2	1.990	1.982
Ir2−N_dpp2	2.024	2.023
Ir2−C_dpp3	1.987	1.981
Ir2−N_dpp3	2.030	2.035
Ir2-O1 (trans to C_dpp3)	2.120	2.113
Ir2-O2	2.127	2.125

Table S2. The coordination bond lengths (in angstrom) in DFT optimized ground state (S_0) and T_1 state geometries of **$Ir_3(dpp)_4(acac)_3$**.

**Parameter**	Ground state (S_0)	state T_1
Bonds (Å)		
Ir1−C_dpp1	1.988	1.984
Ir1−N_dpp1	2.029	2.033
Ir1−C_dpp2	1.990	1.989
Ir1−N_dpp2	2.022	2.023
Ir1-O1 (trans to C_dpp1)	2.121	2.117
Ir1-O2	2.127	2.128
Ir2−C_dpp2	1.988	1.980
Ir2−N_dpp2	2.023	2.024
Ir2−C_dpp3	1.988	1.980
Ir2−N_dpp3	2.023	2.024
Ir2-O1 (trans to C_dpp3)	2.127	2.122
Ir2-O2	2.127	2.122
Ir3−C_dpp3	1.990	1.989
Ir3−N_dpp3	2.022	2.023
Ir3−C_dpp4	1.988	1.984
Ir3−N_dpp4	2.030	2.033
Ir3-O1 (trans to C_dpp4)	2.121	2.117
Ir3-O2	2.127	2.128

Table S3. DFT calculated frontier orbital energies and atomic contributions of complex of **$Ir_2(dpp)_3(acac)_2$** in the **T_1 state optimized geometry** resulting from the Mulliken population analysis.

**Orbitals**	**Energy, eV**	**Contribution (Mulliken) (%)**						
		**Ir1**	**Ir2**	**acac1**	**acac2**	**dppm1**	**dppm2**	**dppm3**
**LUMO+4**	−2.396	1	1	0	0	40	1	57
**LUMO+3**	−2.545	1	1	0	0	10	76	12
**LUMO+2**	−2.971	2	3	0	0	36	2	57
**LUMO+1**	−2.980	1	1	0	0	58	1	37
**LUMO**	−3.148	4	4	0	0	1	90	1
**HOMO**	−5.150	22	22	4	4	11	24	11
**HOMO−1**	−5.211	23	23	3	3	24	21	14
**HOMO−2**	−5.424	19	17	25	22	3	11	3
**HOMO−3**	−5.427	16	18	25	28	5	16	7
**HOMO−4**	−5.901	32	29	4	4	8	16	7
**HOMO−5**	−5.914	28	31	2	2	9	18	9

Table S4. DFT calculated frontier orbital energies and atomic contributions of complex of **$Ir_3(dpp)_4(acac)_3$** in the **T_1 state optimized geometry** resulting from the Mulliken population analysis.

Orbitals	Energy, eV	Contribution (Mulliken), (%)									
		Ir1	Ir2	Ir3	acac1	acac2	acac3	dppm1	dppm2	dppm3	dppm4
**LUMO+4**	−2.591	1	1	1	0	0	0	4	44	45	5
**LUMO+3**	−2.958	0	0	4	0	0	0	0	0	1	95
**LUMO+2**	−2.961	4	0	0	0	0	0	95	1	0	0
**LUMO+1**	−3.087	2	4	2	0	0	0	0	46	43	0
**LUMO**	−3.144	2	4	2	0	1	0	0	46	46	0
**HOMO**	−5.178	10	24	10	2	2	4	4	20	20	4
**HOMO−1**	−5.214	21	4	21	3	2	3	12	11	11	12
**HOMO−2**	−5.264	16	14	16	2	3	2	10	14	14	9
**HOMO−3**	−5.451	18	1	18	25	2	24	3	3	3	3
**HOMO−4**	−5.472	15	5	15	24	1	24	2	5	5	2
**HOMO−5**	−5.482	2	29	2	1	46	1	2	7	7	2
**HOMO−6**	−5.884	30	0	29	1	1	3	6	12	11	6
**HOMO−7**	−5.923	28	8	29	2	1	2	7	8	8	7
**HOMO−8**	−5.979	4	45	4	2	3	2	5	15	15	5

Table S5. TD-DFT calculated lowest triplet and singlet states of **$Ir_2(dpp)_3(acac)_2$** in the **T_1 state optimized geometry.**

State, energy (eV)	*f* (oscillator strength)	Contributing transition coefficients*	Character**
triplets			
T_1, 1.948	(triplet)	HOMO→LUMO (0.70)	$M^{Ir1/Ir2}L^{dpp2}CT/LC^{dpp2}/\ L^{dpp1/dpp3}L^{dpp2}CT$
T_2, 2.010	(triplet)	HOMO−1→LUMO (0.70)	$M^{Ir1/Ir2}L^{dpp2}CT/LC^{dpp2}/\ L^{dpp1/dpp3}L^{dpp2}CT$
T_3, 2.136	(triplet)	HOMO→LUMO+1 (0.65)<br>HOMO−1→LUMO+2 (0.24)	$M^{Ir1/Ir2}L^{dpp1/dpp3}CT/LC^{dpp1/dpp3}/$<br>$L^{dpp2}L^{dpp1/dpp3}CT$
singlets			
S_1, 2.068	0.1306	HOMO→LUMO (0.69)	$M^{Ir1/Ir2}L^{dpp2}CT/LC^{dpp2}/\ L^{dpp1/dpp3}L^{dpp2}CT$

S_2, 2.080	0.0006	HOMO−1→LUMO (0.70)	$M^{Ir1/Ir2}L^{dpp2}CT/LC^{dpp2}/ L^{dpp1/dpp3}L^{dpp2}CT$
S_3, 2.182	0.0024	HOMO→LUMO+1 (0.69)	$M^{Ir1/Ir2}L^{dpp1/dpp3}CT/LC^{dpp1/dpp3}/ L^{dpp2}L^{dpp1/dpp3}CT$
S_4, 2.188	0.0158	HOMO→LUMO+2 (0.68)	$M'^{Ir1/Ir2}L^{dpp1/dpp3}CT/LC^{dpp1/dpp3}/ L^{dpp2}L^{dpp1/dpp3}CT$
S_5, 2.261	0.0697	HOMO–1→LUMO+1 (0.67)	$M^{Ir1/Ir2}L^{dpp1/dpp3}CT/LC^{dpp1/dpp3}/ L^{dpp2}L^{dpp1/dpp3}CT$
S_6, 2.271	0.0195	HOMO–1→LUMO+2 (0.68)	$M^{Ir1/Ir2}L^{dpp1/dpp3}CT/LC^{dpp1/dpp3}/ L^{dpp2}L^{dpp1/dpp3}CT$
S_7, 2.298	0.0838	HOMO–3→LUMO (0.70)	$M'^{Ir1/Ir2}L^{dpp2}CT/LC^{dpp2}/ L^{acac1/acac2}L^{dpp2}CT$
S_8, 2.346	0.0807	HOMO–2→LUMO (0.70)	$M'^{Ir1/Ir2}L^{dpp2}CT/LC^{dpp2}/ L^{dpp1/dpp3}L^{dpp2}CT$
S_{16}, 2.801	0.0331	HOMO–4→LUMO (0.52)<br>HOMO→LUMO+5 (−0.41)	$M''^{Ir1/Ir2}L^{dpp2}CT/LC^{dpp2}$
S_{17}, 2.804	0.0911	HOMO–5→LUMO (0.63)<br>HOMO–2→LUMO+6 (0.15)	$M''^{Ir1/Ir2}L^{dpp2}CT/LC^{dpp2}$

*Square of the coefficient multiplied by two gives percentage contribution of the transition to formation of the excited state, e.g., $0.68^2 \times 2 = 0.92$ (92 %).

**MLCT – Metal (M) to Ligand (L) Charge Transfer. LC – Ligand Centered. LLCT – Ligand to Ligand Charge Transfer. M, M' and M'' indicate major contribution of d-orbitals of different angular momentum orientations, e.g., d_{xy}, d_{xz} and d_{yz}.

Table S6. TD-DFT calculated lowest triplet and singlet states of **$Ir_3(dpp)_4(acac)_3$** in the **T_1 state optimized geometry**.

State, energy (eV)	*f* (oscillator strength)	Contributing transition coefficients*	Character**
		triplets	
T_1, 1.982	(triplet)	HOMO→LUMO (0.69)	$M^{Ir1/Ir2/Ir3}L^{dpp2/dpp3}CT/LC^{dpp2/dpp3}$
T_2, 2.028	(triplet)	HOMO−1→LUMO (0.57)<br>HOMO→LUMO+1 (−0.41)	$M^{Ir1/Ir3}L^{dpp2/dpp3}CT/LC^{dpp2/dpp3}/L^{dpp1/dpp4}L^{dpp2/dpp3}CT$
T_3, 2.059	(triplet)	HOMO→LUMO+1 (0.56)<br>HOMO−1→LUMO (0.39)	$M^{Ir1/Ir2/Ir3}L^{dpp2/dpp3}CT/LC^{dpp2/dpp3}/L^{dpp1/dpp4}L^{dpp2/dpp3}CT$
T_4, 2.071	(triplet)	HOMO→LUMO+1 (0.56)<br>HOMO−1→LUMO+1 (0.41)	$M^{Ir1/Ir2/Ir3}L^{dpp2/dpp3}CT/LC^{dpp2/dpp3}/L^{dpp1/dpp4}L^{dpp2/dpp3}CT$
T_5, 2.110	(triplet)	HOMO−2→LUMO (−0.40)<br>HOMO−1→LUMO+1 (0.56)	$M^{Ir1/Ir2/Ir3}L^{dpp2/dpp3}CT/LC^{dpp2/dpp3}/L^{dpp1/dpp4}L^{dpp2/dpp3}CT$
		singlets	
S_1, 2.074	0.0115	HOMO→LUMO (0.63)<br>HOMO−1→LUMO+1 (0.26)	$M^{Ir1/Ir2/Ir3}L^{dpp2/dpp3}CT/LC^{dpp2/dpp3}$
S_2, 2.082	0.0127	HOMO−1→LUMO (0.64)<br>HOMO→LUMO+1 (0.29)	$M^{Ir1/Ir2/Ir3}L^{dpp2/dpp3}CT/LC^{dpp2/dpp3}/L^{dpp1/dpp4}L^{dpp2/dpp3}CT$
S_3, 2.132	0.2681	HOMO−1→LUMO (−0.29)<br>HOMO→LUMO+1 (0.63)	$M^{Ir1/Ir2/Ir3}L^{dpp2/dpp3}CT/LC^{dpp2/dpp3}/L^{dpp1/dpp4}L^{dpp2/dpp3}CT$
S_4, 2.152	0.0011	HOMO–2→LUMO (0.68)<br>HOMO→LUMO (0.17)	$M^{Ir1/Ir2/Ir3}L^{dpp2/dpp3}CT/LC^{dpp2/dpp3}/L^{dpp1/dpp4}L^{dpp2/dpp3}CT$
S_5, 2.168	0.0188	HOMO–1→LUMO+1 (0.65)<br>HOMO→LUMO (−0.22)	$M^{Ir1/Ir2/Ir3}L^{dpp2/dpp3}CT/LC^{dpp2/dpp3}/L^{dpp1/dpp4}L^{dpp2/dpp3}CT$
S_6, 2.204	0.0186	HOMO–3→LUMO+1 (0.69)	$M'^{Ir1/Ir3}L^{dpp2/dpp3}CT/L^{acac1/acac3}L^{dpp2/dpp3}CT$
S_7, 2.221	0.0021	HOMO→LUMO+2 (0.69)	$M^{Ir1/Ir2/Ir3}L^{dpp1}CT/L^{dpp2/dpp3}L^{dpp1}CT$
S_8, 2.223	0.0018	HOMO→LUMO+3 (0.69)	$M^{Ir1/Ir2/Ir3}L^{dpp4}CT/L^{dpp2/dpp3}L^{dpp4}CT$
S_{11}, 2.319	0.1170	HOMO–3→LUMO (0.64)<br>HOMO–2→LUMO+2 (0.20)<br>HOMO–2→LUMO+3 (0.17)	$M'^{Ir1/Ir3}L^{dpp2/dpp3}CT/L^{acac1/acac3}L^{dpp2/dpp3}CT$

S_{14}, 2.341	0.0012	HOMO–4→LUMO (0.65)<br>HOMO–5→LUMO+1 (0.18)<br>HOMO–3→LUMO+1 (0.17)	$M'^{Ir1/Ir3}L^{dpp2/dpp3}CT$/<br>$L^{acac1/acac3}L^{dpp2/dpp3}CT$
S_{15}, 2.364	0.0904	HOMO–5→LUMO (0.61)<br>HOMO–4→LUMO+1 (0.34)	$M'^{Ir2}L^{dpp2/dpp3}CT$/<br>$L^{acac2}L^{dpp2/dpp3}CT$
S_{30}, 2.764	0.0754	HOMO–6→LUMO (0.64)	$M''^{Ir1/Ir3}L^{dpp2/dpp3}CT/LC^{dpp2/dpp3}$
S_{32}, 2.791	0.0058	HOMO–7→LUMO (−0.34)<br>HOMO→LUMO+6 (0.56)	$M''^{Ir1/Ir3}L^{dpp2/dpp3}CT/LC^{dpp2/dpp3}$
S_{33}, 2.795	0.0040	HOMO–7→LUMO (0.44)<br>HOMO→LUMO+6 (0.38)	$M''^{Ir1/Ir3}L^{dpp2/dpp3}CT/LC^{dpp2/dpp3}$
S_{38}, 2.865	0.0058	HOMO–8→LUMO (−0.44)<br>HOMO–3→LUMO+4 (0.49)	$M''^{Ir2}L^{dpp2/dpp3}CT/LC^{dpp2/dpp3}$
S_{40}, 2.880	0.0025	HOMO–8→LUMO (0.40)<br>HOMO–3→LUMO+4 (0.46)	$M''^{Ir2}L^{dpp2/dpp3}CT/LC^{dpp2/dpp3}$

*Square of the coefficient multiplied by two gives percentage contribution of the transition to formation of the excited state, e.g., $0.68^2 \times 2 = 0.92$ (92 %).

**MLCT – Metal (M) to Ligand (L) Charge Transfer. LC – Ligand Centered. LLCT – Ligand to Ligand Charge Transfer. M, M’ and M’’ indicate major contribution of d-orbitals of different angular momentum orientations, e.g., d_{xy}, d_{xz} and d_{yz}.

Table S7. DFT optimized ground state (S_0) geometry of ΛΔ-isomer of **$Ir_2(dpp)_3(acac)_2$** in cartesian (XYZ) coordinates.

8	-2.816858000	-0.009374000	-2.512168000
6	-2.379553000	0.999203000	-3.103660000
6	-2.240518000	0.857077000	-4.584495000
6	-2.034974000	2.224428000	-2.515254000
6	-2.064394000	2.536754000	-1.146498000
6	-1.790792000	3.945191000	-0.733327000
8	-2.322796000	1.748471000	-0.215530000
1	-3.199024000	0.524090000	-5.020657000
1	-1.514188000	0.055142000	-4.809325000
1	-1.917702000	1.781888000	-5.088833000
1	-1.730173000	3.028841000	-3.195931000
1	-2.740162000	4.418075000	-0.420135000
1	-1.348704000	4.560802000	-1.533280000
1	-1.134159000	3.956924000	0.153009000
8	2.194634000	1.549314000	0.579041000
6	1.807717000	2.645843000	0.127545000
6	1.524835000	3.691998000	1.155641000
6	1.602496000	2.955459000	-1.225393000
6	1.827179000	2.100453000	-2.316356000
6	1.449240000	2.570774000	-3.682623000
8	2.335621000	0.963031000	-2.266488000
1	1.234800000	4.664956000	0.727870000
1	2.416643000	3.830827000	1.791891000
1	0.723716000	3.341500000	1.831611000
1	1.210602000	3.954318000	-1.451724000
1	1.065504000	3.603467000	-3.702853000
1	0.673494000	1.897541000	-4.092617000
1	2.316014000	2.489606000	-4.362355000
6	7.131984000	1.905185000	0.168984000
7	6.416145000	2.237724000	-0.908331000
6	6.653577000	0.953837000	1.062085000
6	5.421575000	0.359155000	0.815953000
7	4.732016000	0.731687000	-0.286117000
6	5.270443000	1.647476000	-1.080502000
6	4.769152000	-0.658397000	1.601046000
6	5.323519000	-1.266450000	2.730329000
6	3.509150000	-1.064260000	1.113119000
6	2.865982000	-2.109078000	1.779091000
6	3.425001000	-2.757495000	2.875290000
6	4.662473000	-2.299653000	3.353690000
6	8.417812000	2.595080000	0.339353000
6	9.301527000	2.308113000	1.382627000
6	10.498257000	2.983515000	1.505175000
6	10.879432000	3.977153000	0.599560000
6	9.994169000	4.257141000	-0.438997000
6	8.792634000	3.583665000	-0.569418000
6	2.770511000	-3.967078000	3.532918000
6	2.522919000	-3.681446000	5.007459000
6	1.448677000	-4.322905000	2.880408000
6	3.706795000	-5.162255000	3.397870000
6	12.210194000	4.696062000	0.776715000
6	12.448895000	5.727387000	-0.309362000
6	13.340477000	3.675587000	0.729053000
1	7.215773000	0.667209000	1.956966000
1	4.674444000	1.910017000	-1.970861000
1	6.300777000	-0.943497000	3.120806000
1	1.892439000	-2.450239000	1.397155000
1	5.129566000	-2.779749000	4.225625000
1	9.062594000	1.533434000	2.123730000

1	11.162064000	2.721437000	2.342346000
1	10.240401000	5.027741000	-1.181176000
1	2.057801000	-4.555753000	5.502718000
1	3.453621000	-3.452947000	5.557927000
1	1.842616000	-2.820044000	5.140714000
1	0.989286000	-5.187916000	3.394194000
1	0.720806000	-3.491622000	2.931580000
1	1.567811000	-4.600246000	1.816161000
1	3.254750000	-6.066123000	3.849965000
1	3.920533000	-5.388778000	2.336711000
1	4.677227000	-4.996070000	3.899999000
6	12.218930000	5.403538000	2.125913000
1	13.422950000	6.225339000	-0.150717000
1	12.478109000	5.276660000	-1.318784000
1	11.678812000	6.520983000	-0.314120000
1	14.321270000	4.172720000	0.854393000
1	13.257717000	2.915384000	1.526861000
1	13.360080000	3.137237000	-0.236749000
1	13.176920000	5.935079000	2.282679000
1	11.406620000	6.150534000	2.197327000
1	12.094388000	4.701828000	2.970617000
77	2.904283000	-0.113299000	-0.526390000
1	8.108291000	3.817818000	-1.394792000
6	-1.231714000	-2.407596000	-1.031989000
7	-1.207698000	-1.130030000	-0.568458000
6	-0.037437000	-2.995610000	-1.421532000
6	1.145892000	-2.282854000	-1.308938000
7	1.114276000	-1.053104000	-0.730344000
6	-0.049062000	-0.529204000	-0.411333000
6	2.453930000	-2.679788000	-1.761349000
6	2.729686000	-3.880824000	-2.414983000
6	3.478813000	-1.730172000	-1.535320000
6	4.754035000	-2.051020000	-1.989301000
6	5.048777000	-3.246815000	-2.644044000
6	4.008747000	-4.160736000	-2.849181000
6	-2.545616000	-2.993688000	-1.056909000
6	-2.807408000	-4.329830000	-1.372856000
6	-4.093421000	-4.813236000	-1.313758000
6	-5.161172000	-3.985742000	-0.929531000
6	-4.884363000	-2.658165000	-0.622358000
6	-3.595513000	-2.124335000	-0.686136000
6	6.477022000	-3.519650000	-3.103468000
6	6.613551000	-4.866804000	-3.787772000
6	6.901026000	-2.436546000	-4.087371000
6	7.402857000	-3.501158000	-1.893405000
6	-6.567471000	-4.570335000	-0.865079000
6	-7.597627000	-3.544522000	-0.432250000
6	-6.585867000	-5.719488000	0.135133000
1	-0.035411000	-4.002558000	-1.852485000
1	-0.057911000	0.486617000	0.016899000
1	1.931874000	-4.617005000	-2.597331000
1	5.569765000	-1.329250000	-1.815992000
1	4.195739000	-5.113387000	-3.359720000
1	-1.991230000	-5.010164000	-1.660353000
1	-4.275386000	-5.868299000	-1.563727000
1	-5.707578000	-1.996142000	-0.316064000
1	7.660992000	-5.025718000	-4.104286000
1	6.347866000	-5.707544000	-3.120093000
1	5.987522000	-4.943637000	-4.696280000
1	7.936668000	-2.610067000	-4.437923000
1	6.244631000	-2.420147000	-4.977396000
1	6.871659000	-1.426543000	-3.640579000
1	8.448942000	-3.696746000	-2.198457000

1	7.390724000	-2.529009000	-1.368558000
1	7.118367000	-4.274091000	-1.155297000
6	-6.956068000	-5.087742000	-2.244415000
1	-8.601007000	-4.007992000	-0.399167000
1	-7.392803000	-3.142331000	0.577084000
1	-7.659389000	-2.689253000	-1.130382000
1	-7.598192000	-6.162218000	0.203733000
1	-5.892692000	-6.533897000	-0.143111000
1	-6.300533000	-5.377810000	1.147528000
1	-7.975561000	-5.518967000	-2.227503000
1	-6.948394000	-4.276643000	-2.996234000
1	-6.274340000	-5.877902000	-2.607909000
6	-7.289322000	1.770013000	-0.001861000
7	-6.805441000	1.505698000	-1.218309000
6	-6.576932000	1.402751000	1.133667000
6	-5.339843000	0.788235000	0.980655000
7	-4.879013000	0.568626000	-0.271293000
6	-5.643748000	0.926434000	-1.294559000
6	-4.461326000	0.310421000	2.019039000
6	-4.712085000	0.432938000	3.384665000
6	-3.284392000	-0.324453000	1.555836000
6	-2.416092000	-0.835644000	2.514959000
6	-2.641765000	-0.705703000	3.885463000
6	-3.810181000	-0.060081000	4.305027000
6	-8.592678000	2.444955000	0.051440000
6	-9.135893000	2.947860000	1.232177000
6	-10.366177000	3.582029000	1.241886000
6	-11.111546000	3.743234000	0.075880000
6	-10.557897000	3.240421000	-1.104444000
6	-9.331238000	2.610283000	-1.123179000
6	-1.621865000	-1.263610000	4.871736000
6	-1.955183000	-0.904738000	6.308091000
6	-1.600223000	-2.781391000	4.746907000
6	-0.245032000	-0.693062000	4.550377000
6	-12.468682000	4.433650000	0.046664000
6	-12.394792000	5.640759000	-0.879839000
6	-12.896524000	4.908824000	1.421972000
1	-6.971990000	1.571612000	2.141192000
1	-5.234060000	0.695389000	-2.292684000
1	-5.623158000	0.933836000	3.746573000
1	-1.502545000	-1.352871000	2.174184000
1	-4.025021000	0.065011000	5.373368000
1	-8.585553000	2.867043000	2.179705000
1	-10.745310000	3.965485000	2.197919000
1	-11.106387000	3.344285000	-2.052761000
1	-1.181938000	-1.309267000	6.987111000
1	-1.990312000	0.188932000	6.470509000
1	-2.922062000	-1.328393000	6.637232000
1	-0.812314000	-3.221821000	5.390286000
1	-2.567437000	-3.223779000	5.050999000
1	-1.407434000	-3.111159000	3.710055000
1	0.494538000	-1.012386000	5.309274000
1	0.142435000	-1.014730000	3.564680000
1	-0.255182000	0.412658000	4.548173000
6	-13.514676000	3.455364000	-0.472718000
1	-13.370190000	6.161985000	-0.921633000
1	-11.639804000	6.371347000	-0.534567000
1	-12.130812000	5.361832000	-1.916015000
1	-13.884305000	5.401301000	1.361653000
1	-12.995390000	4.076611000	2.143622000
1	-12.194388000	5.648273000	1.850116000
1	-14.513290000	3.931363000	-0.504701000
1	-13.290597000	3.101243000	-1.495246000

1	-13.590787000	2.561888000	0.174497000
77	-3.030693000	-0.241932000	-0.413210000
1	-8.911555000	2.225483000	-2.061430000

Table S8. DFT optimized ground state (S_0) and T_1 state geometries of ΛΛ-isomer of **$Ir_2(dpp)_3(acac)_2$** in cartesian (XYZ) coordinates.

State S_0				State T_1			
6	-5.132661000	-1.014403000	0.946269000	6	5.112549390	1.023810077	0.936920072
7	-4.737521000	-0.469747000	-0.227672000	7	4.758028362	0.425020032	-0.224759017
6	-6.322871000	-1.730499000	0.981974000	6	6.280443499	1.769441136	0.977323073
6	-7.065175000	-1.856671000	-0.186410000	6	7.057193519	1.864497145	-0.175066013
7	-6.653175000	-1.270843000	-1.314334000	7	6.691906486	1.213565090	-1.286156096
6	-5.530208000	-0.615736000	-1.281679000	6	5.581890424	0.538802041	-1.259740097
6	-8.328015000	-2.602292000	-0.256956000	6	8.296409635	2.642012202	-0.243859019
6	-8.770968000	-3.434350000	0.770333000	6	8.703280687	3.506758266	0.773277056
6	-9.132391000	-2.491783000	-1.394690000	6	9.120471700	2.535363195	-1.369645105
6	-10.327792000	-3.174314000	-1.485470000	6	10.294537789	3.253292248	-1.458901109
6	-10.782467000	-4.004836000	-0.457090000	6	10.710893813	4.117835314	-0.441722034
6	-9.970424000	-4.118797000	0.669948000	6	9.881796733	4.225733322	0.673876053
6	-4.253145000	-0.726527000	2.053174000	6	4.217381323	0.742058054	2.036513158
6	-4.486692000	-1.126809000	3.371623000	6	4.422794337	1.162800090	3.351145255
6	-3.640743000	-0.718794000	4.378109000	6	3.578276274	0.735526054	4.353252334
6	-2.542100000	0.111416000	4.106085000	6	2.505663191	-0.129439010	4.085004311
6	-2.302633000	0.470665000	2.782801000	6	2.282715175	-0.500821038	2.762952212
6	-3.128452000	0.066287000	1.731571000	6	3.114380236	-0.085194006	1.720564129
6	-12.108371000	-4.739667000	-0.602357000	6	12.014832911	4.891042371	-0.585553047
6	-12.433870000	-5.575389000	0.620639000	6	12.291002950	5.771463464	0.618228048
6	-13.223815000	-3.721381000	-0.802862000	6	13.165749982	3.904000297	-0.736695054
6	-12.036083000	-5.661105000	-1.813609000	6	11.936642898	5.775379429	-1.823870138
6	-1.660047000	0.584326000	5.255204000	6	1.645121128	-0.633313050	5.236584399
6	-0.967591000	-0.624775000	5.870578000	6	0.947168071	0.552862043	5.888898426
6	-2.519333000	1.271513000	6.308952000	6	2.532770195	-1.328692099	6.261669454
1	-6.664211000	-2.162048000	1.928963000	1	6.581403481	2.249723169	1.914512147
1	-5.186578000	-0.118877000	-2.204275000	1	5.273597405	-0.001673000	-2.170692166
1	-8.164282000	-3.577953000	1.675066000	1	8.083641593	3.647972278	1.669441130
1	-10.930627000	-3.052415000	-2.397824000	1	10.912349846	3.132847241	-2.361472180
1	-10.269886000	-4.765446000	1.504694000	1	10.151071751	4.895759372	1.500571113
1	-5.350673000	-1.760613000	3.622688000	1	5.266171403	1.821330140	3.607018277
1	-3.847689000	-1.041883000	5.408337000	1	3.770591288	1.070472080	5.382763407
1	-1.443759000	1.118585000	2.553909000	1	1.445790113	-1.173168091	2.525588190
1	-13.403956000	-6.086379000	0.480896000	1	13.244011011	6.313623495	0.478253036
1	-11.679548000	-6.362375000	0.806676000	1	11.505362858	6.534596484	0.771374061
1	-12.520448000	-4.963903000	1.538107000	1	12.385567930	5.188857395	1.553486118
1	-14.200336000	-4.230634000	-0.910334000	1	14.127325105	4.440756338	-0.845852065
1	-13.300688000	-3.029934000	0.056959000	1	13.250738018	3.242841246	0.145891011
1	-13.076465000	-3.106295000	-1.709035000	1	13.052269985	3.256146249	-1.624792123
1	-12.989259000	-6.207982000	-1.944080000	1	12.874885008	6.347666473	-1.953717149
1	-11.845768000	-5.109383000	-2.752103000	1	11.780466922	5.192294396	-2.749593212
1	-11.232357000	-6.412855000	-1.703337000	1	11.108383822	6.504555515	-1.749102134
6	-0.604710000	1.569679000	4.791721000	6	0.597428043	-1.623445123	4.766592365
1	-0.309732000	-0.318117000	6.707166000	1	0.305478024	0.218407017	6.727076506
1	-1.687490000	-1.361498000	6.272499000	1	1.662546125	1.289103100	6.299475465
1	-0.343105000	-1.148805000	5.122087000	1	0.306080024	1.086770083	5.162321394
1	-1.890044000	1.636966000	7.142780000	1	1.924078148	-1.716649133	7.100510540
1	-3.053682000	2.143852000	5.888373000	1	3.070007237	-2.186278167	5.815410470
1	-3.277865000	0.598788000	6.747803000	1	3.290913253	-0.653063051	6.697294516
1	0.015095000	1.891906000	5.649257000	1	-0.015565001	-1.961609150	5.622708405
1	0.078430000	1.130526000	4.041398000	1	-0.091726007	-1.182516091	4.022733309
1	-1.049980000	2.479883000	4.348396000	1	1.048904080	-2.524325193	4.311035330

8	-2.003731000	-1.410995000	-0.567224000	8	2.044778155	1.370455106	-0.599306046
6	-1.609331000	-1.829930000	-1.678080000	6	1.616720122	1.790808139	-1.697600128
6	-0.769993000	-3.061848000	-1.642954000	6	0.767971060	3.013888233	-1.639912124
6	-1.851870000	-1.233297000	-2.924644000	6	1.842071142	1.196969092	-2.946911226
6	-2.503268000	-0.014837000	-3.168010000	6	2.516509192	-0.009511001	-3.182114241
6	-2.770164000	0.378522000	-4.583713000	6	2.790404213	-0.417138032	-4.590864349
1	-0.655106000	-3.541385000	-2.627576000	1	0.635384049	3.498839266	-2.619598201
1	-1.178877000	-3.788571000	-0.919897000	1	1.179411091	3.738937287	-0.916829070
1	0.240983000	-2.785875000	-1.280343000	1	-0.235918018	2.725467206	-1.266886096
1	-1.508244000	-1.789343000	-3.805298000	1	1.486647115	1.747243135	-3.825980292
1	-3.835458000	0.189718000	-4.816261000	1	3.856787293	-0.230935017	-4.819360370
1	-2.163773000	-0.179821000	-5.315812000	1	2.186571167	0.134254010	-5.330044388
1	-2.610445000	1.462159000	-4.716093000	1	2.631139202	-1.501982117	-4.713339359
8	-2.912864000	0.784993000	-2.301249000	8	2.944756226	-0.785573061	-2.302901175
8	2.024103000	-1.392721000	0.591018000	8	-2.050309158	1.357655102	0.629321048
6	1.633083000	-1.802465000	1.706490000	6	-1.624505123	1.763961135	1.733749131
6	0.802749000	-3.040761000	1.684974000	6	-0.780002058	2.990692230	1.693934131
6	1.871626000	-1.190585000	2.946371000	6	-1.848743141	1.151770089	2.974353226
6	2.514680000	0.034876000	3.176553000	6	-2.519381190	-0.060127004	3.191888245
6	2.778442000	0.445311000	4.587979000	6	-2.792632213	-0.488758037	4.594511348
8	2.919144000	0.828011000	2.301270000	8	-2.944716225	-0.824869065	2.301424178
1	-0.209962000	-2.776329000	1.318666000	1	0.225331017	2.711008205	1.318233101
1	0.690801000	-3.509891000	2.674928000	1	-0.650304048	3.462578262	2.680364207
1	1.217370000	-3.772670000	0.970453000	1	-1.193172091	3.724145285	0.980378077
1	1.531816000	-1.739397000	3.833020000	1	-1.495695115	1.690650131	3.861396295
1	2.611428000	1.529228000	4.708546000	1	-2.630865200	-1.574888120	4.701275361
1	3.844862000	0.266109000	4.822927000	1	-3.859487293	-0.308333024	4.825445369
1	2.175447000	-0.109156000	5.325813000	1	-2.190250166	0.053327004	5.341720419
6	7.073545000	-1.833290000	0.218063000	6	-7.062053536	1.849674144	0.212454016
7	6.665279000	-1.228043000	1.337081000	7	-6.695668513	1.183013089	1.314009102
6	6.331018000	-1.722096000	-0.951630000	6	-6.284906474	1.772754135	-0.940940073
6	5.142248000	-1.003081000	-0.925411000	6	-5.116102392	1.027850081	-0.911624072
7	4.748927000	-0.441411000	0.240948000	7	-4.760843363	0.412459031	0.241067019
6	5.543582000	-0.571361000	1.295739000	6	-5.584916418	0.510201039	1.277621097
6	4.262069000	-0.730066000	-2.035504000	6	-4.220655322	0.763565056	-2.015251154
6	4.496358000	-1.145835000	-3.349014000	6	-4.426812337	1.202911091	-3.323685253
6	3.136281000	0.065011000	-1.723436000	6	-3.116355236	-0.066553005	-1.711481130
6	2.309519000	0.454942000	-2.779425000	6	-2.284200173	-0.465884036	-2.759804211
6	2.549390000	0.079731000	-4.098229000	6	-2.507981193	-0.075990006	-4.076392311
6	3.649700000	-0.751521000	-4.360302000	6	-3.581839272	0.791267061	-4.331951330
6	8.331882000	-2.585233000	0.298402000	6	-8.302221642	2.624557200	0.292162022
6	8.760681000	-3.447886000	-0.713979000	6	-8.714253653	3.498471265	-0.719551056
6	9.951042000	-4.137125000	-0.600166000	6	-9.890489739	4.210575320	-0.606416045
6	10.775361000	-4.003592000	0.520995000	6	-10.719330804	4.093233314	0.513770039
6	10.339072000	-3.144642000	1.528217000	6	-10.301051802	3.223819245	1.520091118
6	9.144360000	-2.453884000	1.424014000	6	-9.121582708	2.508135189	1.416434111
6	1.666937000	0.537339000	-5.253218000	6	-1.647240128	-0.562449041	-5.235287401
6	0.981896000	-0.680704000	-5.859178000	6	-0.951427075	0.633669049	-5.871524426
6	0.605316000	1.521031000	-4.800769000	6	-0.597821048	-1.557452122	-4.779651365
6	2.525051000	1.219452000	-6.311207000	6	-2.534451195	-1.244814097	-6.269468469
6	12.081371000	-4.782862000	0.600182000	6	-12.009102927	4.898933372	0.592495048
6	12.831715000	-4.506255000	1.888910000	6	-12.767175953	4.634777356	1.879387145
6	12.970165000	-4.386205000	-0.571882000	6	-12.904476970	4.523924348	-0.581900046
1	6.670850000	-2.168234000	-1.892402000	1	-6.585584511	2.267004172	-1.870895141
1	5.201966000	-0.059275000	2.210768000	1	-5.275881403	-0.043171003	2.180572165
1	5.361621000	-1.780870000	-3.592450000	1	-5.271165402	1.863852141	-3.569962270
1	1.449658000	1.104331000	-2.558448000	1	-1.446194113	-1.140292086	-2.532274192
1	3.857483000	-1.086195000	-5.386670000	1	-3.774877291	1.140627087	-5.356532408
1	8.146024000	-3.608693000	-1.610356000	1	-8.098615615	3.647517281	-1.617104124
1	10.243476000	-4.811096000	-1.418822000	1	-10.169070763	4.889730371	-1.425756108
1	10.944722000	-3.000670000	2.432503000	1	-10.910486824	3.091139237	2.423657185
1	0.324235000	-0.384851000	-6.699789000	1	-0.309565023	0.311765024	-6.714468487
1	1.706149000	-1.417430000	-6.253117000	1	-1.668124130	1.374347104	-6.271681488

1	0.358390000	-1.201177000	-5.107387000	1	-0.310862024	1.158555089	-5.137961392
1	-0.015285000	1.831135000	-5.662190000	1	0.015370001	-1.882604146	-5.640650425
1	-0.076066000	1.084885000	-4.047083000	1	0.090925007	-1.125953087	-4.029908307
1	1.044423000	2.438244000	-4.365866000	1	-1.047703081	-2.465353188	-4.336630331
1	1.895898000	1.574219000	-7.149745000	1	-1.925688148	-1.620130124	-7.113994559
1	3.053999000	2.098208000	-5.897214000	1	-3.070196232	-2.109318163	-5.834916420
1	3.288082000	0.546616000	-6.742057000	1	-3.293817251	-0.564250043	-6.695169499
6	11.777829000	-6.274145000	0.528038000	6	-11.675324906	6.384097469	0.524476040
1	13.771870000	-5.087075000	1.909677000	1	-13.695378037	5.234595402	1.900110144
1	13.108061000	-3.440784000	1.995422000	1	-13.065292984	3.574898271	1.982879149
1	12.252608000	-4.798036000	2.784700000	1	-12.183529908	4.912218378	2.776774211
1	13.928328000	-4.938797000	-0.538292000	1	-13.850414059	5.097351388	-0.549369044
1	12.502721000	-4.604913000	-1.549185000	1	-12.430471953	4.733785360	-1.558010118
1	13.207212000	-3.306050000	-0.552972000	1	-13.164992994	3.449142262	-0.565387044
1	12.711974000	-6.864574000	0.585605000	1	-12.597438964	6.993445547	0.579852046
1	11.127743000	-6.596098000	1.362888000	1	-11.021714855	6.690905494	1.362289102
1	11.271845000	-6.557340000	-0.412803000	1	-11.160130848	6.658740514	-0.413938032
77	2.944167000	0.486519000	0.209057000	77	-2.952452225	-0.520025040	0.210053016
1	8.819158000	-1.783975000	2.230378000	1	-8.812961668	1.829777137	2.222141170
6	-1.203305000	2.781770000	-0.028009000	6	1.205008093	-2.826046216	-0.036168003
7	-1.159871000	1.425182000	-0.068166000	7	1.159725089	-1.443925112	-0.089257007
6	-0.005601000	3.484199000	-0.014282000	6	0.002477000	-3.517435268	-0.021558002
6	1.196454000	2.789413000	0.007042000	6	-1.201387092	-2.828750217	0.003816000
7	1.161448000	1.433124000	0.062062000	7	-1.158954088	-1.447532111	0.078264006
6	0.002690000	0.817880000	0.000269000	6	-0.000214000	-0.843028063	-0.000882000
6	2.534938000	3.322903000	-0.055334000	6	-2.533545196	-3.349841256	-0.069344005
6	2.834050000	4.682116000	-0.151867000	6	-2.864022219	-4.703584358	-0.178593014
6	3.572437000	2.354985000	-0.061028000	6	-3.582922276	-2.378856182	-0.066313005
6	4.876939000	2.820710000	-0.190988000	6	-4.894464375	-2.821405215	-0.194592015
6	5.193761000	4.176932000	-0.288480000	6	-5.233424401	-4.172213320	-0.303025023
6	4.144235000	5.102747000	-0.261394000	6	-4.183977321	-5.100486387	-0.287223022
6	-2.545091000	3.307693000	0.027887000	6	2.538208195	-3.345519254	0.029012002
6	-2.852766000	4.666019000	0.108751000	6	2.871480221	-4.700158358	0.116769009
6	-4.165659000	5.079753000	0.212026000	6	4.192231322	-5.096016390	0.219386017
6	-5.209488000	4.147852000	0.248275000	6	5.239719398	-4.165934319	0.250464019
6	-4.884112000	2.792616000	0.166577000	6	4.897968375	-2.814271213	0.163395012
6	-3.576628000	2.333573000	0.043362000	6	3.585592272	-2.372478183	0.041491003
6	6.652589000	4.601201000	-0.419870000	6	-6.696136517	-4.580348348	-0.430360033
6	6.806988000	6.107883000	-0.506851000	6	-6.863211534	-6.085193446	-0.526881040
6	7.428034000	4.108678000	0.795808000	6	-7.464369589	-4.089783311	0.790946063
6	7.239441000	3.984081000	-1.683304000	6	-7.285991558	-3.950309302	-1.686337130
6	-6.671092000	4.564400000	0.373208000	6	6.703240510	-4.572979348	0.371836028
6	-7.255555000	3.957854000	1.642853000	6	7.291395569	-3.961711304	1.637847126
6	-7.441882000	4.053390000	-0.837775000	6	7.470810575	-4.061518308	-0.841286065
1	-0.009023000	4.579697000	-0.020442000	1	0.003537000	-4.614790352	-0.030093002
1	0.006070000	-0.283234000	0.006458000	1	-0.001337000	0.259302020	0.007584001
1	2.031019000	5.434790000	-0.143991000	1	-2.075770156	-5.471449439	-0.183095014
1	5.696712000	2.083077000	-0.214153000	1	-5.700656426	-2.068785159	-0.207359016
1	4.347345000	6.178342000	-0.333271000	1	-4.396766336	-6.174716478	-0.367131028
1	-2.054398000	5.423506000	0.093259000	1	2.084840159	-5.469646426	0.108692008
1	-4.375411000	6.154827000	0.271565000	1	4.407244336	-6.170947466	0.282092021
1	-5.699456000	2.050351000	0.197102000	1	5.702546435	-2.060239158	0.188482014
1	7.876582000	6.371995000	-0.599128000	1	-7.934984591	-6.341700475	-0.616343045
1	6.292077000	6.534647000	-1.387680000	1	-6.354779461	-6.509548514	-1.412777110
1	6.423597000	6.623730000	0.393173000	1	-6.479098490	-6.609970480	0.367817028
1	8.492066000	4.405486000	0.725559000	1	-8.533142634	-4.369990332	0.721078055
1	7.023725000	4.535103000	1.732965000	1	-7.063581535	-4.529692348	1.723423131
1	7.401355000	3.008562000	0.896112000	1	-7.422221573	-2.990785227	0.901216071
1	8.299810000	4.277722000	-1.804074000	1	-8.352091624	-4.226203322	-1.800582138
1	7.205035000	2.879832000	-1.668357000	1	-7.234408577	-2.846516216	-1.666553129
1	6.695264000	4.318351000	-2.586449000	1	-6.753812498	-4.288117330	-2.595371196
6	-6.835037000	6.070970000	0.443004000	6	6.873447515	-6.078809451	0.444549034
1	-8.317761000	4.246522000	1.759393000	1	8.357989621	-4.237301322	1.748162136

1	-7.214659000	2.853728000	1.640208000	1	7.237669579	-2.857843218	1.635517127
1	-6.714195000	4.305240000	2.542734000	1	6.759520522	-4.314890328	2.541202195
1	-8.508061000	4.343364000	-0.771693000	1	8.540174659	-4.340454330	-0.775487059
1	-7.039641000	4.472648000	-1.779051000	1	7.071271539	-4.487509343	-1.780733138
1	-7.407253000	2.952490000	-0.926280000	1	7.426273589	-2.961007227	-0.934187073
1	-7.906386000	6.329410000	0.530891000	1	7.945729588	-6.334459478	0.530306042
1	-6.324006000	6.510935000	1.319589000	1	6.365631511	-6.518229508	1.323424102
1	-6.453652000	6.578944000	-0.462336000	1	6.490713508	-6.590145489	-0.458485035
77	-2.936789000	0.466148000	-0.205570000	77	2.951375228	-0.511039039	-0.207329016
1	-8.795549000	-1.845515000	-2.215276000	1	8.815288672	1.863433144	-2.181944168

Table S8. DFT optimized ground state (S_0) and T_1 state geometries of ΛΛΛ-isomer of **$Ir_3(dpp)_4(acac)_3$** in cartesian (XYZ) coordinate.

**State S_0**				**State T_1**			
8	4.818040000	0.080606000	-1.406232000	8	4.857971000	0.050802000	-1.419583000
6	4.812974000	-0.751492000	-2.340355000	6	4.848741000	-0.779112000	-2.355504000
6	3.955698000	-0.405629000	-3.510502000	6	3.992087000	-0.429661000	-3.524625000
6	5.496500000	-1.976522000	-2.364540000	6	5.530840000	-2.004174000	-2.379081000
6	6.254383000	-2.545330000	-1.329890000	6	6.291880000	-2.560917000	-1.340387000
6	6.982410000	-3.820774000	-1.599768000	6	7.024945000	-3.835741000	-1.595815000
8	6.401302000	-2.076060000	-0.182533000	8	6.436823000	-2.076465000	-0.199385000
1	2.896005000	-0.579002000	-3.232904000	1	2.932653000	-0.610118000	-3.250085000
1	4.172226000	-1.010445000	-4.405086000	1	4.212760000	-1.028343000	-4.422321000
1	4.047273000	0.666866000	-3.754414000	1	4.078776000	0.644658000	-3.761935000
1	5.441092000	-2.546629000	-3.299683000	1	5.479804000	-2.576396000	-3.312982000
1	6.837000000	-4.525496000	-0.762570000	1	6.868700000	-4.537204000	-0.757819000
1	8.068569000	-3.616925000	-1.645386000	1	8.111346000	-3.631213000	-1.627064000
1	6.685378000	-4.304485000	-2.544602000	1	6.739655000	-4.323039000	-2.542299000
6	9.340299000	2.352865000	-0.945857000	6	9.325544000	2.382674000	-0.933765000
7	9.402009000	1.021068000	-1.031479000	7	9.409106000	1.051850000	-1.012870000
6	8.235676000	2.970653000	-0.371570000	6	8.208129000	2.984079000	-0.365484000
6	7.185813000	2.180725000	0.080410000	6	7.170942000	2.177761000	0.085098000
7	7.277242000	0.838129000	-0.059404000	7	7.284552000	0.836916000	-0.048112000
6	8.385746000	0.339045000	-0.591246000	6	8.403279000	0.354047000	-0.573178000
6	5.984174000	2.622476000	0.745855000	6	5.959318000	2.602563000	0.745307000
6	5.705471000	3.953011000	1.070276000	6	5.662297000	3.927313000	1.073784000
6	5.098531000	1.589429000	1.126405000	6	5.084493000	1.557787000	1.121503000
6	3.974259000	1.946705000	1.875105000	6	3.953853000	1.895573000	1.870459000
6	3.705900000	3.261739000	2.244872000	6	3.672970000	3.204241000	2.250762000
6	4.582928000	4.264956000	1.803557000	6	4.536569000	4.220220000	1.810928000
6	10.492375000	3.100242000	-1.464384000	6	10.465208000	3.146834000	-1.454562000
6	10.456120000	4.478989000	-1.688455000	6	10.415349000	4.528563000	-1.657878000
6	11.561671000	5.146193000	-2.176100000	6	11.509057000	5.211856000	-2.149284000
6	12.757621000	4.480379000	-2.460622000	6	12.705978000	4.559842000	-2.460273000
6	12.784184000	3.104574000	-2.239016000	6	12.746513000	3.181221000	-2.259081000
6	11.677651000	2.426992000	-1.757440000	6	11.652379000	2.487833000	-1.772016000
6	2.513599000	3.643345000	3.114156000	6	2.486386000	3.565375000	3.136304000
6	1.583031000	4.542170000	2.310701000	6	1.544363000	4.475389000	2.359253000
6	1.737647000	2.427234000	3.581808000	6	1.721822000	2.336862000	3.590515000
6	3.007078000	4.392755000	4.345853000	6	2.989934000	4.295194000	4.375956000
6	13.952514000	5.259950000	-2.993186000	6	13.887478000	5.356974000	-2.996869000
6	15.156711000	4.367572000	-3.225242000	6	15.091272000	4.475722000	-3.270268000
6	14.335864000	6.336339000	-1.985296000	6	14.283177000	6.412574000	-1.971934000
1	8.185991000	4.057825000	-0.247746000	1	8.137698000	4.070660000	-0.247936000
1	8.427414000	-0.761483000	-0.650042000	1	8.463968000	-0.745845000	-0.627248000
1	6.383511000	4.762137000	0.758978000	1	6.328819000	4.747209000	0.766401000
1	3.291643000	1.150894000	2.208220000	1	3.284916000	1.087745000	2.201304000
1	4.391350000	5.317333000	2.057470000	1	4.332665000	5.268770000	2.071194000
1	9.537075000	5.051950000	-1.502838000	1	9.495343000	5.092281000	-1.450806000

1	11.483281000	6.230119000	-2.346505000	1	11.420529000	6.297742000	-2.300866000
1	13.696082000	2.529106000	-2.444912000	1	13.659907000	2.616326000	-2.486633000
1	0.697500000	4.828840000	2.910677000	1	0.664540000	4.746964000	2.974319000
1	2.076770000	5.477321000	1.988104000	1	2.030397000	5.418907000	2.049749000
1	1.222741000	4.028957000	1.399046000	1	1.176189000	3.979006000	1.441742000
1	2.356254000	1.749324000	4.199079000	1	2.349669000	1.653901000	4.192695000
1	2.156922000	4.662550000	5.001122000	1	2.145081000	4.553540000	5.042531000
1	3.704128000	3.774660000	4.942122000	1	3.692620000	3.668371000	4.956158000
1	3.531227000	5.331747000	4.092239000	1	3.511070000	5.238667000	4.132770000
6	13.571781000	5.912972000	-4.316047000	6	13.480499000	6.036923000	-4.298268000
1	16.003043000	4.968161000	-3.605674000	1	15.926725000	5.088181000	-3.655720000
1	15.501329000	3.875540000	-2.296732000	1	15.457275000	3.967432000	-2.358900000
1	14.959048000	3.578405000	-3.974224000	1	14.882005000	3.700510000	-4.030599000
1	15.202838000	6.919535000	-2.349642000	1	15.140982000	7.008106000	-2.338009000
1	13.515879000	7.053996000	-1.800906000	1	13.462977000	7.121651000	-1.757611000
1	14.615689000	5.896510000	-1.009823000	1	14.581940000	5.952566000	-1.011529000
1	14.424114000	6.487709000	-4.725669000	1	14.322865000	6.624651000	-4.709959000
1	13.283363000	5.158812000	-5.071886000	1	13.182815000	5.297935000	-5.065421000
1	12.725625000	6.616170000	-4.211443000	1	12.633041000	6.733480000	-4.164130000
77	5.650654000	-0.224122000	0.527604000	77	5.662534000	-0.245009000	0.528314000
1	11.720222000	1.342606000	-1.592086000	1	11.705979000	1.401684000	-1.622731000
6	1.833834000	-2.560039000	2.105308000	6	1.825242000	-2.544669000	2.131206000
7	1.786283000	-1.855189000	0.944746000	7	1.792957000	-1.872682000	0.933354000
6	2.996351000	-2.512765000	2.863871000	6	2.976634000	-2.469906000	2.898841000
6	4.087866000	-1.789640000	2.401465000	6	4.082265000	-1.773959000	2.426396000
7	4.000068000	-1.188830000	1.187114000	7	4.010373000	-1.219174000	1.172153000
6	2.864085000	-1.230346000	0.530030000	6	2.882291000	-1.277792000	0.509417000
6	5.342988000	-1.543429000	3.067993000	6	5.328188000	-1.515402000	3.092335000
6	5.666739000	-2.027481000	4.335673000	6	5.653707000	-1.963249000	4.374957000
6	6.249770000	-0.710835000	2.361863000	6	6.256407000	-0.715039000	2.367947000
6	7.442911000	-0.385112000	2.998812000	6	7.456831000	-0.391484000	2.988548000
6	7.780567000	-0.857309000	4.268554000	6	7.794714000	-0.833185000	4.270899000
6	6.869586000	-1.693181000	4.924843000	6	6.866074000	-1.631101000	4.948735000
6	0.630043000	-3.308864000	2.365278000	6	0.623959000	-3.290212000	2.389675000
6	0.460334000	-4.173395000	3.450784000	6	0.428937000	-4.132200000	3.488488000
6	-0.687453000	-4.924062000	3.564025000	6	-0.722664000	-4.882405000	3.592487000
6	-1.700146000	-4.849882000	2.594326000	6	-1.727574000	-4.837836000	2.611039000
6	-1.530147000	-3.963395000	1.534255000	6	-1.541732000	-3.970494000	1.538291000
6	-0.388147000	-3.173870000	1.389726000	6	-0.394277000	-3.188470000	1.399173000
6	9.110970000	-0.450194000	4.892333000	6	9.136146000	-0.437028000	4.877206000
6	9.314029000	-1.064695000	6.264514000	6	9.329478000	-1.014063000	6.266989000
6	10.249312000	-0.907654000	3.988720000	6	10.261596000	-0.947380000	3.985502000
6	9.150273000	1.066411000	5.033960000	6	9.213909000	1.081822000	4.973163000
6	-2.936300000	-5.728683000	2.738526000	6	-2.958053000	-5.722600000	2.757930000
6	-3.864191000	-5.603266000	1.546010000	6	-3.885175000	-5.600804000	1.564632000
6	-3.690411000	-5.307174000	3.993220000	6	-3.716932000	-5.309431000	4.012690000
1	3.049092000	-3.037452000	3.824156000	1	3.015328000	-2.959429000	3.879412000
1	2.809140000	-0.698739000	-0.432240000	1	2.839314000	-0.778911000	-0.471562000
1	4.968333000	-2.679383000	4.882227000	1	4.947655000	-2.585772000	4.945460000
1	8.154147000	0.274543000	2.473665000	1	8.175095000	0.241994000	2.441093000
1	7.095666000	-2.090956000	5.921840000	1	7.086217000	-2.004468000	5.957081000
1	1.243535000	-4.271894000	4.217700000	1	1.196518000	-4.217195000	4.272065000
1	-0.795839000	-5.599130000	4.424816000	1	-0.840971000	-5.540041000	4.466349000
1	-2.315330000	-3.888268000	0.767238000	1	-2.311828000	-3.905030000	0.755576000
1	10.287988000	-0.746256000	6.679576000	1	10.311788000	-0.706331000	6.670603000
1	8.537537000	-0.750592000	6.986647000	1	8.562092000	-0.660310000	6.980587000
1	9.321967000	-2.170252000	6.234230000	1	9.310880000	-2.119936000	6.270192000
1	11.227810000	-0.626892000	4.423253000	1	11.248560000	-0.670768000	4.403794000
1	10.245096000	-2.006040000	3.857852000	1	10.234969000	-2.049346000	3.892666000
1	10.197374000	-0.455609000	2.981817000	1	10.211561000	-0.529876000	2.963476000
1	10.108622000	1.391043000	5.482732000	1	10.182396000	1.397873000	5.406514000
1	9.051282000	1.583311000	4.062401000	1	9.121709000	1.570931000	3.986523000
1	8.335128000	1.432590000	5.685902000	1	8.411271000	1.487259000	5.617409000

6	-2.515026000	-7.187662000	2.861782000	6	-2.525843000	-7.178703000	2.880774000
1	-4.747952000	-6.253800000	1.684618000	1	-4.769244000	-6.250790000	1.703630000
1	-4.235827000	-4.570491000	1.413236000	1	-4.255335000	-4.567624000	1.428957000
1	-3.375743000	-5.909897000	0.602346000	1	-3.396382000	-5.910424000	0.622128000
1	-4.606652000	-5.915093000	4.124902000	1	-4.627059000	-5.926523000	4.144114000
1	-3.082587000	-5.427140000	4.909030000	1	-3.109543000	-5.424247000	4.929519000
1	-3.993976000	-4.244344000	3.936849000	1	-4.030615000	-4.249687000	3.957060000
1	-3.404546000	-7.839705000	2.953093000	1	-3.408996000	-7.838434000	2.979525000
1	-1.947851000	-7.521297000	1.972748000	1	-1.961181000	-7.509064000	1.988956000
1	-1.883246000	-7.377537000	3.747932000	1	-1.886411000	-7.361001000	3.763409000
1	1.333443000	1.837353000	2.738647000	1	1.316386000	1.755807000	2.741796000
1	0.878347000	2.742749000	4.202584000	1	0.865339000	2.636738000	4.222751000
6	-1.837108000	-2.552237000	-2.113702000	6	-1.826566000	-2.530989000	-2.143926000
7	-1.787006000	-1.849805000	-0.951809000	7	-1.792995000	-1.865051000	-0.942732000
6	-3.000719000	-2.502093000	-2.870392000	6	-2.978040000	-2.450663000	-2.910891000
6	-4.090709000	-1.778683000	-2.404850000	6	-4.082539000	-1.755513000	-2.434651000
7	-4.000404000	-1.180504000	-1.189390000	7	-4.009519000	-1.207140000	-1.177643000
6	-2.863494000	-1.224687000	-0.534087000	6	-2.881335000	-1.270690000	-0.515527000
6	-5.346469000	-1.529503000	-3.069040000	6	-5.328217000	-1.491715000	-3.099002000
6	-5.672946000	-2.011065000	-4.336972000	6	-5.654848000	-1.932924000	-4.383636000
6	-6.250813000	-0.696567000	-2.360221000	6	-6.254898000	-0.693254000	-2.370564000
6	-7.444403000	-0.367918000	-2.994817000	6	-7.454951000	-0.364757000	-2.989276000
6	-7.784704000	-0.837469000	-4.264840000	6	-7.793938000	-0.799801000	-4.273615000
6	-6.876107000	-1.673819000	-4.923813000	6	-6.866820000	-1.596031000	-4.955492000
6	-0.634553000	-3.301904000	-2.376846000	6	-0.626508000	-3.277087000	-2.406360000
6	-0.467022000	-4.163429000	-3.465053000	6	-0.432985000	-4.113785000	-3.509471000
6	0.679858000	-4.914910000	-3.581827000	6	0.717366000	-4.865335000	-3.617413000
6	1.693745000	-4.844540000	-2.613116000	6	1.722441000	-4.827427000	-2.635854000
6	1.525896000	-3.961020000	-1.550190000	6	1.538198000	-3.965160000	-1.558743000
6	0.384836000	-3.170811000	-1.401962000	6	0.392033000	-3.181984000	-1.415500000
6	-9.115254000	-0.427001000	-4.886118000	6	-9.134878000	-0.398530000	-4.877638000
6	-9.321318000	-1.038978000	-6.258980000	6	-9.329548000	-0.968586000	-6.270113000
6	-10.253188000	-0.883800000	-3.981670000	6	-10.260925000	-0.911289000	-3.988071000
6	-9.151979000	1.089892000	-5.025324000	6	-9.210173000	1.120886000	-4.966312000
6	2.929139000	-5.723683000	-2.761814000	6	2.951378000	-5.713561000	-2.787352000
6	3.682763000	-5.296915000	-4.015033000	6	3.711113000	-5.294974000	-4.039799000
6	2.506960000	-7.181843000	-2.891408000	6	2.516665000	-7.168232000	-2.918081000
1	-3.055513000	-3.024723000	-3.831687000	1	-3.017700000	-2.935135000	-3.893931000
1	-2.806457000	-0.695161000	0.429206000	1	-2.837318000	-0.776654000	0.467852000
1	-4.976469000	-2.663317000	-4.885564000	1	-4.950005000	-2.553932000	-4.957282000
1	-8.153795000	0.292011000	-2.467522000	1	-8.172011000	0.267316000	-2.438622000
1	-7.104346000	-2.069634000	-5.921097000	1	-7.087856000	-1.964254000	-5.965534000
1	-1.251155000	-4.258775000	-4.231414000	1	-1.200808000	-4.193571000	-4.293357000
1	0.786667000	-5.587410000	-4.444809000	1	0.834484000	-5.518725000	-4.494612000
1	2.312143000	-3.888825000	-0.783968000	1	2.308516000	-3.904887000	-0.775824000
1	-10.295278000	-0.718109000	-6.672167000	1	-10.311461000	-0.657293000	-6.671963000
1	-8.545268000	-0.725139000	-6.981701000	1	-8.561777000	-0.612687000	-6.982229000
1	-9.331224000	-2.144565000	-6.230419000	1	-9.312785000	-2.074459000	-6.278628000
1	-11.231759000	-0.600446000	-4.414354000	1	-11.247548000	-0.631057000	-4.404761000
1	-10.250897000	-1.982411000	-3.852657000	1	-10.236082000	-2.013728000	-3.900509000
1	-10.198986000	-0.433545000	-2.974089000	1	-10.209928000	-0.498755000	-2.964074000
1	-10.110333000	1.416995000	-5.472289000	1	-10.178223000	1.440589000	-5.397959000
1	-9.050743000	1.605084000	-4.053088000	1	-9.117008000	1.605114000	-3.977360000
1	-8.337037000	1.455603000	-5.677781000	1	-8.406989000	1.528094000	-5.608756000
6	3.857707000	-5.604256000	-1.569221000	6	3.878545000	-5.599834000	-1.593298000
1	4.598858000	-5.904389000	-4.149761000	1	4.620149000	-5.912971000	-4.174496000
1	3.074421000	-5.412864000	-4.931026000	1	3.103578000	-5.403751000	-4.957269000
1	3.986472000	-4.234359000	-3.954278000	1	4.026678000	-4.236107000	-3.978419000
1	3.396106000	-7.833934000	-2.985922000	1	3.398699000	-7.828935000	-3.020280000
1	1.939927000	-7.519114000	-2.003658000	1	1.951328000	-7.502423000	-2.028119000
1	1.874822000	-7.367569000	-3.778175000	1	1.877037000	-7.344704000	-3.801756000
1	4.740586000	-6.255326000	-1.710907000	1	4.761400000	-6.250755000	-1.735602000
1	4.230673000	-4.572506000	-1.432447000	1	4.250649000	-4.568102000	-1.452095000

1	3.369268000	-5.914147000	-0.626613000	1	3.389003000	-5.913506000	-0.652522000
77	-0.000064000	-1.810992000	-0.004107000	77	0.000038000	-1.849574000	-0.004749000
8	0.665776000	-0.283197000	-1.326045000	8	0.652548000	-0.298991000	-1.298742000
8	-0.662082000	-0.284713000	1.321494000	8	-0.649774000	-0.304300000	1.296896000
6	-0.656591000	0.940909000	1.071076000	6	-0.663085000	0.924738000	1.061797000
6	-1.452489000	1.798053000	1.995774000	6	-1.482296000	1.757764000	1.985607000
1	-2.528445000	1.668198000	1.757287000	1	-2.553266000	1.610954000	1.733532000
1	-1.214853000	2.871238000	1.909647000	1	-1.261927000	2.835305000	1.909892000
1	-1.319915000	1.469409000	3.041543000	1	-1.354973000	1.422811000	3.029956000
6	0.004602000	1.560720000	0.000095000	6	0.004452000	1.556924000	0.004411000
6	0.663856000	0.941780000	-1.072584000	6	0.669677000	0.928674000	-1.056765000
6	1.462010000	1.798911000	-1.995341000	6	1.490922000	1.764425000	-1.976293000
1	1.226721000	2.872460000	-1.907294000	1	1.273267000	2.842106000	-1.894956000
1	1.329152000	1.472442000	-3.041755000	1	1.362689000	1.435201000	-3.022350000
1	2.537595000	1.666277000	-1.756643000	1	2.561557000	1.613670000	-1.725080000
1	0.006086000	2.657102000	0.001453000	1	0.006095000	2.652741000	0.007468000
6	-7.177880000	2.195357000	-0.079032000	6	-7.162555000	2.191458000	-0.075074000
7	-7.270932000	0.853071000	0.063677000	7	-7.279183000	0.850195000	0.052547000
6	-8.225591000	2.987717000	0.373245000	6	-8.198015000	2.998063000	0.378559000
6	-9.329215000	2.373009000	0.952879000	6	-9.316558000	2.396603000	0.944583000
7	-9.392116000	1.041557000	1.042362000	7	-9.403519000	1.065731000	1.017691000
6	-8.378283000	0.356904000	0.600319000	6	-8.399019000	0.367510000	0.575115000
6	-10.478824000	3.122534000	1.474045000	6	-10.454461000	3.160995000	1.468806000
6	-10.446028000	4.500388000	1.683392000	6	-10.398655000	4.536616000	1.688269000
6	-11.661167000	2.446000000	1.786722000	6	-11.648709000	2.502297000	1.774598000
6	-12.758750000	3.126843000	2.271537000	6	-12.735078000	3.198158000	2.263017000
6	-12.736217000	4.509138000	2.478996000	6	-12.689237000	4.578346000	2.480945000
6	-11.550118000	5.175449000	2.175855000	6	-11.491669000	5.226710000	2.184102000
6	-5.976330000	2.634206000	-0.746632000	6	-5.950011000	2.616363000	-0.733551000
6	-5.695526000	3.963882000	-1.072725000	6	-5.649947000	3.941879000	-1.056082000
6	-4.572247000	4.273236000	-1.806005000	6	-4.523468000	4.235504000	-1.791809000
6	-3.696512000	3.268161000	-2.245582000	6	-3.662155000	3.219535000	-2.236100000
6	-3.967239000	1.953977000	-1.874559000	6	-3.946146000	1.909818000	-1.861784000
6	-5.092365000	1.599311000	-1.125895000	6	-5.077584000	1.571302000	-1.114375000
6	-13.972036000	5.218202000	3.016430000	6	-13.913411000	5.304329000	3.022747000
6	-13.750012000	6.710270000	3.173753000	6	-13.665437000	6.790953000	3.192881000
6	-15.129565000	5.001006000	2.049804000	6	-15.073309000	5.115210000	2.052962000
6	-14.328884000	4.635719000	4.378234000	6	-14.282114000	4.716880000	4.379337000
6	-2.502614000	3.646876000	-3.113933000	6	-2.474478000	3.581893000	-3.119669000
6	-1.570722000	4.543515000	-2.309555000	6	-1.530530000	4.486060000	-2.338141000
6	-2.993056000	4.397321000	-4.346204000	6	-2.975903000	4.318635000	-4.356082000
1	-8.174320000	4.074475000	0.246820000	1	-8.126131000	4.084998000	0.264962000
1	-8.421463000	-0.743410000	0.661709000	1	-8.462240000	-0.732468000	0.624255000
1	-9.531073000	5.074671000	1.482267000	1	-9.474049000	5.096947000	1.492268000
1	-13.670922000	2.554179000	2.496603000	1	-13.657231000	2.639604000	2.482634000
1	-11.468970000	6.259067000	2.330464000	1	-11.392395000	6.307636000	2.346472000
1	-6.372338000	4.774452000	-0.762524000	1	-6.314608000	4.761908000	-0.745048000
1	-4.378908000	5.325027000	-2.061003000	1	-4.317100000	5.284744000	-2.047304000
1	-3.285626000	1.156847000	-2.206523000	1	-3.279073000	1.101960000	-2.196269000
1	-14.667392000	7.188605000	3.562720000	1	-14.574757000	7.282295000	3.584515000
1	-12.937720000	6.942914000	3.887258000	1	-12.850385000	7.003121000	3.909614000
1	-13.510677000	7.204178000	2.213625000	1	-13.415981000	7.288813000	2.237387000
1	-16.043643000	5.504748000	2.417573000	1	-15.978507000	5.632673000	2.423560000
1	-14.902926000	5.410976000	1.047850000	1	-14.837935000	5.528341000	1.054369000
1	-15.376749000	3.931659000	1.920815000	1	-15.339693000	4.051480000	1.915883000
1	-15.227479000	5.132461000	4.790912000	1	-15.171607000	5.227071000	4.795270000
1	-14.548638000	3.553732000	4.330118000	1	-14.522144000	3.639683000	4.321990000
1	-13.507355000	4.773945000	5.105736000	1	-13.458820000	4.833710000	5.108566000
6	-1.729026000	2.428929000	-3.580774000	6	-1.712721000	2.353658000	-3.579335000
1	-0.683999000	4.828253000	-2.908703000	1	-0.649879000	4.758392000	-2.951692000
1	-2.062695000	5.479716000	-1.987291000	1	-2.014441000	5.429283000	-2.024435000
1	-1.212446000	4.029318000	-1.397647000	1	-1.163835000	3.984535000	-1.422839000
1	-2.141584000	4.664823000	-5.000701000	1	-2.130240000	4.577992000	-5.021236000

1	-3.691087000	3.780876000	-4.943034000	1	-3.679963000	3.696226000	-4.939362000
1	-3.515037000	5.337712000	-4.093322000	1	-3.494788000	5.262256000	-4.108681000
1	-0.868791000	2.742418000	-4.201281000	1	-0.855355000	2.654413000	-4.209957000
1	-1.326364000	1.838503000	-2.737251000	1	-1.308880000	1.767664000	-2.733241000
1	-2.348781000	1.752086000	-4.198076000	1	-2.342031000	1.675007000	-4.184855000
77	-5.647896000	-0.213009000	-0.526334000	77	-5.659611000	-0.232722000	-0.528961000
1	-11.701645000	1.359875000	1.633378000	1	-11.707477000	1.418210000	1.613164000
8	-4.812041000	0.088166000	1.406737000	8	-4.853890000	0.052901000	1.419992000
8	-6.401986000	-2.063731000	0.183608000	8	-6.437731000	-2.065643000	0.191055000
6	-6.253233000	-2.535078000	1.329868000	6	-6.292859000	-2.555702000	1.329668000
6	-6.983100000	-3.809515000	1.599603000	6	-7.028210000	-3.830272000	1.579744000
1	-8.068549000	-3.603166000	1.650805000	1	-8.114163000	-3.623718000	1.613186000
1	-6.683012000	-4.296400000	2.541859000	1	-6.742879000	-4.322677000	2.523572000
1	-6.843209000	-4.512365000	0.759919000	1	-6.874273000	-4.528005000	0.738217000
6	-5.492213000	-1.969041000	2.363730000	6	-5.529972000	-2.005275000	2.370373000
6	-4.807154000	-0.744861000	2.340038000	6	-4.845730000	-0.781319000	2.352091000
6	-3.948383000	-0.401288000	3.509762000	6	-3.987974000	-0.438743000	3.522435000
1	-4.165214000	-1.006459000	4.404024000	1	-4.209252000	-1.041210000	4.417443000
1	-4.038085000	0.671156000	3.754557000	1	-4.072769000	0.634608000	3.764759000
1	-2.889211000	-0.576187000	3.231233000	1	-2.928973000	-0.619685000	3.246565000
1	-5.436116000	-2.540321000	3.298113000	1	-5.479380000	-2.581898000	3.301587000

References.

(1) Kozhevnikov, V. N.; Durrant, M. C.; Williams, J. A. G. Highly Luminescent Mixed-Metal Pt(II)/Ir(III) Complexes: Bis-Cyclometalation of 4,6-Diphenylpyrimidine As a Versatile Route to Rigid Multimetallic Assemblies. *Inorg. Chem.* **2011,** *50*, 6304-6313.

(2) Frisch, M. J.; Trucks, G. W.; Schlegel, H. B.; Scuseria, G. E.; Robb, M. A.; Cheeseman, J. R.; Scalmani, G.; Barone, V.; Mennucci, B.; Petersson, G. A.; Nakatsuji, H.; Caricato, M.; Li, X.; Hratchian, H. P.; Izmaylov, A. F.; Bloino, J.; Zheng, G.; Sonnenberg, J. L.; Hada, M.; Ehara, M.; Toyota, K.; Fukuda, R.; Hasegawa, J.; Ishida, M.; Nakajima, T.; Honda, Y.; Kitao, O.; Nakai, H.; Vreven, T.; Montgomery Jr., J. A.; Peralta, J. E.; Ogliaro, F.; Bearpark, M. J.; Heyd, J.; Brothers, E. N.; Kudin, K. N.; Staroverov, V. N.; Kobayashi, R.; Normand, J.; Raghavachari, K.; Rendell, A. P.; Burant, J. C.; Iyengar, S. S.; Tomasi, J.; Cossi, M.; Rega, N.; Millam, N. J.; Klene, M.; Knox, J. E.; Cross, J. B.; Bakken, V.; Adamo, C.; Jaramillo, J.; Gomperts, R.; Stratmann, R. E.; Yazyev, O.; Austin, A. J.; Cammi, R.; Pomelli, C.; Ochterski, J. W.; Martin, R. L.; Morokuma, K.; Zakrzewski, V. G.; Voth, G. A.; Salvador, P.; Dannenberg, J. J.; Dapprich, S.; Daniels, A. D.; Farkas, Ö.; Foresman, J. B.; Ortiz, J. V.; Cioslowski, J.; Fox, D. J. *Gaussian 09*. Gaussian, Inc.: Wallingford, CT, USA, 2009.

(3) Peverati, R.; Truhlar, D. G. Performance of the M11 and M11-L density functionals for calculations of electronic excitation energies by adiabatic time-dependent density functional theory. *Phys. Chem. Chem. Phys.* **2012,** *14*, 11363-11370.

(4) Weigend, F.; Ahlrichs, R. Balanced basis sets of split valence, triple zeta valence and quadruple zeta valence quality for H to Rn: Design and assessment of accuracy. *Phys. Chem. Chem. Phys.* **2005,** *7*, 3297-3305.

(5) Cossi, M.; Rega, N.; Scalmani, G.; Barone, V. Energies, structures, and electronic properties of molecules in solution with the C-PCM solvation model. *J. Comput. Chem.* **2003,** *24*, 669-681.
